# Nutraceuticals for the Management of Cardiovascular Health: A Mechanistic and Evidence-Based Review Focusing on Atherosclerosis and Hypertension

**DOI:** 10.3390/nu18142226

**Published:** 2026-07-08

**Authors:** Phaniendra Alugoju, Charanraj Goud Alladi, Nyshadham S. N. Chaitanya, Luxitaa Goenka, Kishoree K. Kumaree, Sudarshan SJ, Krishnaswamy Venkataramanasamy Krishnasami Duraisamy, Naeem Ullah

**Affiliations:** 1 Department of Biophysics, Faculty of Science, Palacky University, 779 00 Olomouc, Czech Republic; 2 Institute of Molecular and Translational Medicine, Faculty of Medicine and Dentistry, Palacký University and University Hospital Olomouc, Hněvotínská, 779 00 Olomouc, Czech Republic; charan.raj002@gmail.com; 3 Department of Animal Biology, School of Life Sciences, University of Hyderabad, Hyderabad 500046, Telangana, India; nsainagachaitanya8@gmail.com; 4 Department of Medical Oncology, Jawaharlal Institute of Postgraduate Medical Education and Research (JIPMER), Puducherry 605006, India; 5 College of Public Health Science, Chulalongkorn University, Bangkok 10330, Thailand; kishoree.k3@gmail.com; 6 Department of Physiology, University of Tennessee Health Science Center, Memphis, TN 38163, USA; sjsudharshan@gmail.com; 7 Institute of Fisheries Biotechnology, Tamilnadu Dr. J. Jayalalithaa Fisheries University, Chennai 603103, Tamil Nadu, India; krishna130592@gmail.com; 8 Department of Molecular Biology, Umeå University, 901 87 Umeå, Sweden; naeem.ullah@umu.se

**Keywords:** nutraceuticals, cardiovascular health, atherosclerosis, hypertension

## Abstract

Cardiovascular diseases (CVDs) constitute one of the leading global health burdens, accounting for nearly 50% of all deaths worldwide. Rapid urbanization, lifestyle transitions, psychosocial stress, socioeconomic factors, and changing dietary patterns significantly influence cardiovascular health. Among modifiable risk determinants, atherosclerosis (AS) and hypertension (HTN) are the most critical, interrelated contributors to the onset and progression of CVDs. Nutritional interventions have gained significant global recognition in public health science and clinical practice and are widely regarded as a preventive approach for effective disease management. The goal of the current review is to present evidence from research conducted over the past decade (from 2016 to 2025) supporting the role of nutraceuticals in the management of AS and HTN. This review thoroughly discusses the role of nutraceuticals derived from diverse sources (plants, animals, microorganisms, etc.) from mechanistic aspects to clinical trial endpoints.

## 1. Introduction

Cardiovascular diseases (CVDs) constitute an important group of non-communicable diseases of the heart and blood vessels. The most important CVDs include stroke, ischemic heart disease (also known as coronary heart disease), cerebrovascular disease, peripheral arterial disease, rheumatic heart disease, congenital heart disease, deep vein thrombosis, and pulmonary embolism. CVDs constitute a significant cause of global mortality, and the World Health Organization (WHO) estimates that about 17.9 million deaths occur every year globally due to CVDs [1]. In 2022, it was estimated that about 85% of global CVD-related mortality is due to stroke and heart attack, and about three-quarters of these deaths were reported only in low- and middle-income countries [1]. In northwestern and southern Europe, there is a moderate downward trend in mortality and morbidity due to CVDs [2]. It is estimated that CVDs account for approximately 42.5–45% of all deaths in the World Health Organization’s (WHO) broader European region consisting of 53 states [3,4] and 31–37% of deaths in the European Union (EU) consisting of 27 member states [5,6]. In view of rising global CVD-associated deaths, the European Union’s annual cost of medical care for CVD is about EUR 192 billion [7]. According to statistics, by 2030, 23.6 million people will die from CVDs [2]. These facts are the critical reasons that draw more attention and substantial importance to the topic of CVDs in the field of public health. Particularly, behavioral risk factors, also known as lifestyle factors, including unhealthy food (e.g., excess intake of salt, fats, and sugars), physical inactivity, consumption of tobacco products, and excess alcohol consumption, contribute to the onset and progression of diverse CVDs [8]. These lifestyle-associated risk factors significantly alter various physiological functions causing high blood pressure and elevated levels of blood glucose and lipids that drive the development of atherosclerosis (AS) and hypertension (HTN), which act as interrelated pathologies that contribute to the development of several other CVDs.

Nutraceuticals are broadly defined as food-derived bioactive compounds that provide health benefits beyond basic nutritive and therapeutic value, bridging the gap between nutrition and pharmaceuticals. Globally, lifestyle and dietary patterns have changed dramatically, with a substantial rise in the consumption of junk and processed foods. This shift has contributed to extensive nutritional imbalances and, in turn, an increase in lifestyle-related diseases [9]. Consequently, there is a growing interest in safer, nutrition-based alternative approaches known as nutraceuticals [10]. Increasing preclinical and clinical scientific evidence supports their potential health benefits, particularly cardiovascular health and other physiological functions [11]. It is important to note that lifestyle interventions such as balanced diets, regular exercise, and smoking cessation remain the primary strategies for the prevention and management of cardiovascular health, while nutraceuticals are considered complementary adjunct approaches. While complex dietary patterns such as the Mediterranean diet may exert nutraceutical-like effects, this review focuses specifically on individual nutraceutical classes with particular emphasis on AS and HTN, the two major drivers of CVDs. HTN was prioritized alongside AS because it is one of the most prevalent and modifiable cardiovascular risk factors, directly accelerating vascular injury and atherosclerotic progression. Rather than independent pathologies, they represent two deeply interlinked mechanisms underlying the development of most CVDs, making them crucial targets for nutraceutical interventions.

### 1.1. Atherosclerosis (AS)

AS is a chronic inflammatory disease of the arteries. It is the underlying cause of approximately 50% of mortality in Western nations [12]. The global mortality rates associated with CVDs are dominated by AS-associated diseases, particularly coronary artery disease (CAD), ischemic heart disease (IHD), and stroke [13]. The burden of AS is largely evaluated by examining the incidence of CAD, IHD, and stroke, its major clinical manifestations. As of 2020, CAD alone affected 11.5% of the global adult population and caused about 8.9 million deaths. It was estimated that nearly 31.87 million people had IHD globally in 2021, resulting in about 8.99 million deaths. Global IHD cases have increased enormously; in fact, they more than doubled from 1990 to 2021. Of these cases, 43.65% of IHD cases occurred in women, about 56.35% in men, with the highest incidence observed in individuals older than 50 years. It is predicted that IHD incidence will increase by 0.30–fold from 2022 to 2036, with a more pronounced occurrence in women than men [14]. Stroke accounts for roughly one-quarter of all CVD deaths, about half of which are due to ischemic stroke [15]. The global increase in the incidence of AS-related diseases highlights the inadequacy of contemporary preventive strategies and emphasizes the need for more effective lifestyle-based prevention strategies for the management of AS and other associated CVDs [16].

Modifiable (lifestyle) risk factors involved in the etiology of AS include smoking, HTN, diabetes, hypercholesterolemia (elevated low-density lipoprotein cholesterol, LDL-C), dyslipidemia (elevated LDL-C, reduced high-density lipoprotein cholesterol, HDL-C, and elevated triglycerides, TG), obesity/metabolic syndrome, physical inactivity, unhealthy diet (high saturated fat, low fiber, and excess sodium worsen lipid profile), excessive alcohol intake, and elevated homocysteine [12,17]. Other non-modifiable risk factors include age (male older than 45 years and female older than 55 years), male sex, and a strong family history [12]. Certain genetic conditions such as familial hypercholesterolemia and belonging to higher-risk groups such as Black or South Asian populations further increase susceptibility to AS [12,17,18].

The symptoms of AS do not usually appear in the early stages. As the AS plaques progress, symptoms begin to manifest due to arterial narrowing, which interrupts and reduces blood flow to all vital and peripheral organs, and ultimately contributes to the development of CVDs. Rupture of AS plaques leads to the formation of blood clots in arteries, leading to a partial or complete blockage of blood flow and oxygen supply to vital organs, which in turn triggers a heart attack or stroke. In coronary artery disease (CAD), where the plaque builds up in the coronary arteries, patients may experience symptoms such as chest pain, shortness of breath, or an irregular heartbeat (arrhythmias). Similarly, accumulation of AS plaque in the carotid artery, which supplies blood to the brain, face, and head, leads to carotid artery disease, and patients may experience symptoms such as dizziness, confusion, weakness, vision problems, and headache. Likewise, in peripheral artery disease, where plaques accumulate in the peripheral arteries supplying blood to the peripheral organs (arms, legs, or pelvis) often experience symptoms such as numbness, tingling sensation, and pain. In renal artery stenosis, the buildup of AS plaques in the renal arteries reduces blood flow to the kidneys, leading to symptoms such as high blood pressure, fatigue, nausea, loss of appetite, confusion, or swelling in the legs or feet [19].

Adopting healthy lifestyle interventions such as following a healthy diet, engaging in physical activity, maintaining weight control, discontinuing smoking, and limiting alcohol intake help in the prevention and management of AS-related CVDs. Drugs such as statins (e.g., simvastatin, atorvastatin, and rosuvastatin), lipid-lowering agents (PCSK9 inhibitors such as alirocumab), and cholesterol absorption inhibitors (ezetimibe) reduce LDL-cholesterol. Antihypertensive drugs help control blood pressure, and antiplatelet agents such as low-dose aspirin reduce thrombotic events. In advanced stages, stenting or surgical bypass may be required to restore adequate blood flow [20,21].

### 1.2. Hypertension (HTN) or High Blood Pressure (High BP)

HTN is a condition in which the pressure in blood vessels is too high (140/90 mmHg or higher). It is a major cause of premature deaths worldwide. The number of adults with HTN has increased globally from 650 million in 1990 to 1.4 billion in 2024. As of September 2025, according to WHO reports, about 1.4 billion adults aged 30–79 years had HTN globally, with the majority residing in low- and middle-income countries [22]. In addition to lifestyle factors, both environmental (e.g., air pollution) and non-modifiable factors (e.g., age over 65 years, family history of HTN, and co-existing diseases such as diabetes or kidney disease) increase the risk of developing HTN.

Most hypertensive patients do not exhibit any symptoms, but very high blood pressure (BP) (>180/120 mmHg) can cause severe headaches, blurred vision, dizziness, difficulty breathing, nausea, vomiting, anxiety, abnormal heart rhythms (arrhythmias), etc. It is important to note that, when left untreated, HTN can cause severe cardiovascular complications such as heart attack, heart failure, angina pectoris, coronary heart disease, kidney disease, and stroke. Therefore, preventive measures such as healthy behavioral changes (e.g., consuming a low-salt diet, physical exercise, losing body weight, and quitting smoking) help maintain blood pressure in HTN, consequently reducing the development of CVDs. Currently, many commonly prescribed medicines lower blood pressure by promoting vasorelaxation and can also prevent renal damage in hypertensive patients. Hypertensive drugs that can be used as a monotherapy or combination therapy include angiotensin-converting enzyme (ACE) inhibitors (e.g., captopril, lisinopril, ramipril, and enalapril), angiotensin II receptor blockers (ARBs) (e.g., valsartan, losartan and telmisartan), long-acting calcium channel blockers (CCBs) (e.g., dihydropyridines including amlodipine, nifedipine, and felodipine), and diuretics that promote the elimination of excess water from the body (e.g., hydrochlorothiazide, metolazone, and chlorthalidone).

Thus, HTN and AS constitute two major interrelated secondary risk factors that reinforce each other, subsequently increasing the risk of the onset and progression of several cardiovascular complications. Therefore, there is a growing interest in understanding the pathophysiological mechanisms underlying AS and HTN in public health science, as well as in identifying modifiable, nutraceutical-based approaches for the prevention, treatment, and better management of the associated CVDs. In this context, the present review focuses on the molecular mechanisms implicated in both AS and HTN, and how they are modulated by nutraceutical bioactive compounds of diverse origin as evidenced by the recent in vitro and in vivo studies conducted over the last decade (2016–2025). Additionally, selected clinical studies relevant to antihypertensive and anti-atherosclerotic effects of nutraceuticals are discussed briefly.

## 2. Methods

We conducted a comprehensive literature search in three electronic databases: PubMed, Web of Science, and Scopus, to identify studies published between 1 January 2016 and 31 December 2025. Relevant keywords and Medical Subject Headings (MeSH) terms were identified with the assistance of the heTOP terminology tool (https://www.hetop.eu/hetop/, accessed on 15 December 2025). The search strategy combined terms related to nutraceuticals with cardiovascular outcomes (e.g., atherosclerosis and hypertension). The search was limited to articles published in English. No other filters were applied. We included in vitro, animal, and clinical studies to synthesize mechanistic and clinical evidence regarding the effects of nutraceuticals on AS and HTN.

The general search string used was as follows: (nutraceutic* OR nutraceutical* OR “dietary supplement*” OR “food supplement*” OR “herbal supplement*” OR “functional food*” OR “herb therapy” OR “polyphenol mixture”) AND (hypertension OR hypertensive OR “blood pressure” OR “high blood pressure” OR atherosclerosis OR arteriosclerosis OR “coronary artery disease” OR “endothelial disease” OR “endothelial dysfunction” OR “vascular dysfunction”).

This review is presented as a narrative synthesis rather than a systematic review, and therefore does not include formal inclusion/exclusion criteria, PRISMA flow diagram, or risk-of -bias assessment. Studies were selected based on their relevance to the mechanistic and clinical themes of this review.

## 3. The Pathogenesis of AS: Mechanisms of Initiation, Progression, and Complications

**Figure 1:**
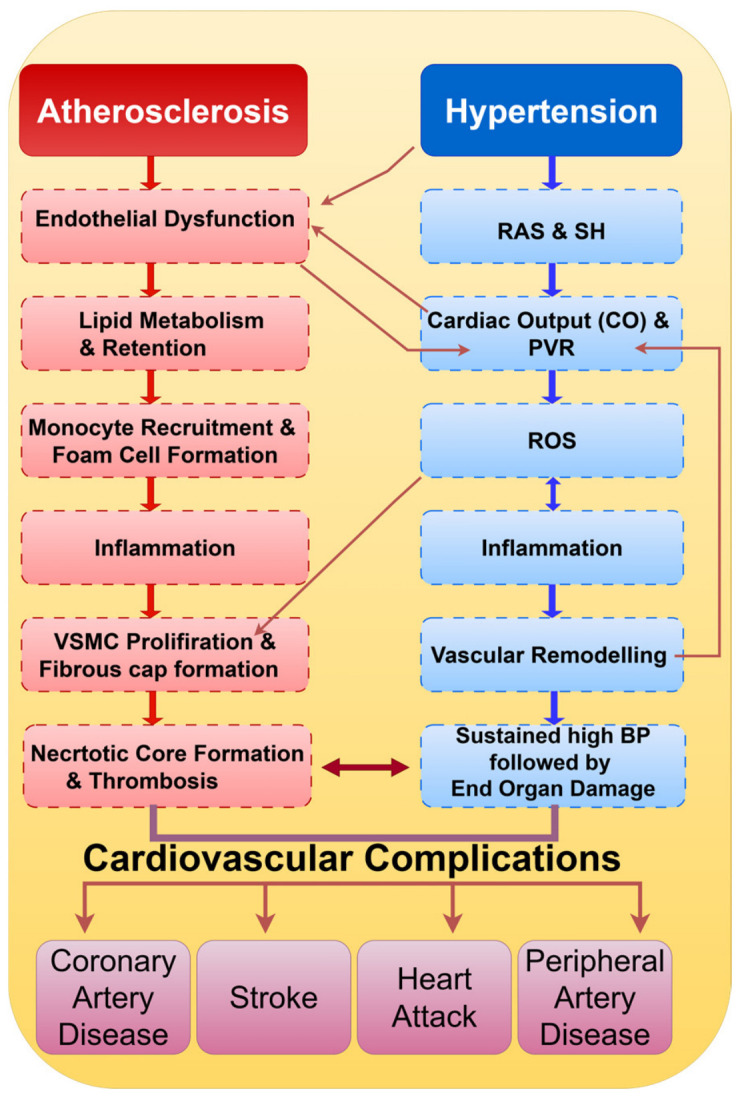
Pathophysiological mechanisms involved in AS and HTN. Abbreviations: VSMC: vascular smooth muscle cell; RAS: renin–angiotensin system; SH: sympathetic hyperactivity; ROS: reactive oxygen species; BP: blood pressure.

AS is a chronic inflammatory disorder of the medium and large arteries and is the primary underlying cause of most CVDs. AS is characterized by the gradual accumulation of lipid-rich plaque within the arterial walls, leading to hardening and narrowing of the arteries, thereby reducing blood flow and increasing the risk of CVDs [23]. This multistage process evolves over decades and often remains clinically silent until it triggers acute thrombotic events. The pathogenesis involves an initial stage of endothelial dysfunction, followed by a cascade of events that include lipid accumulation, immune cell filtration, foam cell formation, smooth muscle cell migration, and ultimately plaque development and vessel narrowing (Figure 1).

### 3.1. Endothelial Dysfunction (ED)

The vascular endothelium serves as a critical barrier and signaling interface between the bloodstream and the arterial wall [24]. Endothelial nitric oxide synthase (eNOS) converts L-arginine into nitric oxide (NO), a vasodilator that promotes smooth muscle cell relaxation and helps prevent atherogenesis and its complications [25]. Endothelial dysfunction (ED) is the earliest measurable deterioration of the vessel wall in atherogenesis. Pathogenesis begins when the endothelial layer transitions from a quiescent state to an activated or dysfunctional phenotype. ED is characterized by reduced NO bioavailability, which impairs vasodilation and promotes platelet aggregation, leukocyte adhesion, and smooth muscle cell proliferation [26,27].

### 3.2. Lipid Metabolism and Lipoprotein Retention

The accumulation and retention of LDL within the arterial wall represents the critical initiating event in AS [28]. Following entry into the intimal extracellular matrix, LDL particles undergo progressive changes, notably oxidation driven by lipoxygenases (e.g., 15-lipoxygenase) and myeloperoxidase (MPO). These oxidized LDL (oxLDL) particles act as potent pro-inflammatory stimuli that drive the formation of early lesions, which eventually contribute to the pathophysiology of AS [29,30]. In addition, apolipoprotein B-100, a major protein component of LDL, undergoes modifications in response to oxidative stress. These modifications impair recognition by the LDL receptor (LDLR), thereby increasing the uptake of LDL particles via non-regulated receptors [31,32].

### 3.3. Monocyte Recruitment and Foam Cell Formation

The differentiation of monocytes into macrophages following their recruitment into the intima represents a critical step in the development of an atherosclerotic lesion [33]. In response to endothelial activation, circulating monocytes are selectively recruited into the intima [34], where they differentiate into macrophages [35]. These macrophages promote the production of cytokines, reactive oxygen species (ROS), and NO, eventually amplifying local inflammation [36]. Moreover, macrophages express a series of receptors (e.g., CD36 and SRA-1) that mediate the internalization of both modified and unmodified LDL [37,38,39]. These lipid-laden (primarily containing oxidized LDL, i.e., oxLDL) macrophages are called foam cells. Beyond serving as lipid reservoirs, foam cells actively propagate inflammation by activating signaling pathways and contributing to plaque growth and instability [29].

### 3.4. Inflammation and Immune Activation

While lipid accumulation and foam cell formation initiate atherosclerotic lesions, sustained disease progression is largely driven by chronic inflammation within the arterial wall. Inflammation actively drives lesion development through complex interactions between innate and adaptive immune responses. In established plaques, macrophages, T cells (particularly Th1 cells), and B cells produce a diverse array of cytokines, chemokines, and reactive mediators that sustain a self-perpetuating inflammatory milieu and accelerate lesion progression [40,41,42,43].

### 3.5. Vascular Smooth Muscle Cell (VSMC) Proliferation and Fibrous Cap Formation

Vascular smooth muscle cell (VSMC) activation begins early in atherogenesis, triggered by signals arising from endothelial dysfunction and subintimal lipid accumulation [44,45]. Following activation, VSMCs migrate from the media into the intima, proliferate, and synthesize extracellular matrix (ECM) components, contributing to the development of the fibrous cap, a collagen-rich structure that covers the lipid core and provides mechanical stability to the plaque [46,47]. Although initial VSMC proliferation supports fibrous cap development, prolonged inflammatory signaling and oxidative stress induce VSMC apoptosis and impair matrix synthesis in advanced lesions [48]. This results in the thinning of the fibrous cap and increased susceptibility to rupture. In addition, matrix-degrading enzymes, particularly MMP-2, MMP-8, and MMP-9, released by activated macrophages and foam cells, further compromise cap integrity by degrading collagen and other ECM components [49]. Thus, the gradual depletion of VSMCs, along with persistent inflammation, shifts the plaque from a stable to a vulnerable state, increasing the risk of thrombosis and acute cardiovascular events.

### 3.6. Necrotic Core Formation and Thrombosis

As atherosclerotic lesions progress, the failure to resolve inflammation and efficiently clear apoptotic foam cells leads to the formation of a necrotic core, a hallmark of advanced plaques [50,51,52]. As the necrotic core expands, it increases mechanical strain on the overlying fibrous cap, thereby increasing plaque susceptibility to rupture [53,54]. Plaque rupture exposes the thrombogenic contents of the necrotic core to circulating blood, leading to thrombus formation [55]. At the same time, recruitment and activation of circulating platelets at the rupture site further accelerate platelet aggregation and accelerate thrombus formation [56].

## 4. Pathophysiological Mechanisms in HTN

According to Irvine Page’s Mosaic theory of HTN, interplay between multiple factors, including genetics, environmental, adaptive, neural, mechanical, and hormonal perturbations, interacts to raise blood pressure in hypertensive patients [57,58,59]. HTN remains a major risk factor for different chronic diseases, including AS, stroke, heart failure, kidney failure, and dementia [60,61]. Based on the presence or absence of an identifiable underlying cause, clinically HTN can also be classified as either primary or secondary [62]. Primary (or essential) HTN is the most common type, accounting for more than 90% of cases with no identifiable cause and is linked to lifestyle factors, age, and genetics [63]. Secondary HTN accounts for about 10% of total cases, and it is caused by identifiable underlying conditions such as renal artery stenosis, hyperthyroidism, Cushing syndrome, etc. [60,64]. Several physiological mechanisms discussed below [65] are involved in the maintenance of normal blood pressure, and any alterations in these mechanisms may contribute to the development of essential HTN (Figure 1).

### 4.1. Cardiac Output (CO) and Peripheral Vascular Resistance (PVR)

A balance between cardiac output (CO) and peripheral vascular resistance (PVR), also known as systemic vascular resistance (SVR), and determined primarily by small arteries and arterioles rather than by large arteries, helps maintain normal blood pressure in humans [65,66]. A normal cardiac output with increased PVR is usually observed in patients with primary (essential) HTN. Among different blood vessels, small arterioles predominantly contribute to PVR because they have a smaller diameter and a high proportion of smooth muscle cells (SMCs), which play a crucial role in regulating vascular constriction. A rise in intracellular calcium levels leads to SMC contraction. However, continued constriction of SMCs can cause vascular remodeling changes, particularly through angiotensin-mediated induction of arteriolar wall thickening, subsequently increasing PVR [67]. It is suggested that during early phases of HTN, PVR remains normal, while blood pressure elevation is attributable to increased cardiac output resulting from sympathetic nervous system (SNS) hyperactivity [68]. As the condition progresses, PVR gradually rises and becomes the dominant factor contributing to sustained HTN.

### 4.2. Renin-Angiotensin System (RAS) Hyperactivity

The circulating *Renin-angiotensin system* (RAS) constitutes one of the predominant mechanisms regulating blood pressure [65]. Reduced blood flow to the glomerulus of the kidney, low salt intake, or sympathetic stimulation causes the secretion of renin from the juxtaglomerular apparatus, which converts angiotensinogen to angiotensin I [65]. Angiotensin I is then rapidly converted by ACE into angiotensin II, a potent vasoconstrictor that elevates blood pressure. Furthermore, renin can stimulate the adrenal cortex to release aldosterone, which promotes sodium and water reabsorption in the kidneys. These events increase blood volume, leading to elevated blood pressure. Although circulating renin and angiotensin II levels are low in the elderly and black population with essential HTN, accumulating scientific evidence indicates that high levels of local non-circulating tissue RAS activity in the kidney, the heart, and the vasculature contribute significantly to blood pressure elevation. This suggests that the rise in blood pressure in these patients with essential HTN is contributed predominantly by non-circulating tissue RAS rather than the classical circulating RAS. Clinical evidence suggests that renal transplant recipients are more likely to develop HTN when they receive kidneys from hypertensive donors, suggesting a critical role of the kidneys in primary HTN and a possible inherited defect in sodium reabsorption [65].

### 4.3. Sympathetic Hyperactivity

Increased activity of the SNS, often referred to as sympathetic hyperactivity, is implicated in the development and progression of several CVDs, including HTN [69]. Sympathetic hyperactivity increases heart rate and myocardial contractility, thereby increasing cardiac output, and also promotes peripheral vasoconstriction, thereby increasing PVR. Additionally, sympathetic hyperactivity in the kidneys induces the release of renin, which in turn promotes sodium and water reabsorption in the kidneys. Together, these events contribute to elevated blood pressure in patients with HTN [69].

### 4.4. Endothelial Dysfunction

Vascular endothelial cells secrete several local vasoactive agents such as vasodilators (e.g., NO) and vasoconstrictors (e.g., endothelin), which play crucial roles in the regulation of blood pressure. Endothelial dysfunction, characterized by impaired NO production, is commonly observed in patients with essential HTN [70]. Endothelin can cause an increase in blood pressure, thereby contributing to the progression of HTN. Endothelin can also increase blood pressure by stimulating local tissue RAS activity.

### 4.5. Reactive Oxygen Species (ROS)

Previous studies have revealed increased activation of ROS-producing enzymes (e.g., NADPH oxidase and nitric oxide synthase (NOS)) in different tissues of hypertensive rats. Excess ROS, including superoxide, can oxidize the cofactor of NOS and inactivate the vasodilator NO [71,72,73]. This loss of NO impairs vasodilation and promotes vasoconstriction, altering vascular diameter and vascular reactivity. These events ultimately elevate PVR and contribute to an increase in blood pressure in HTN. Additionally, ROS can also enhance renal sodium reabsorption, leading to increased blood volume and cardiac output, contributing to a rise in blood pressure.

### 4.6. Inflammatory and Immune Mechanisms

It is well documented that both inflammatory and immune cells play a crucial role in the development and progression of HTN. Accumulation of inflammatory cells has been reported in the blood vessels and kidneys of hypertensive patients and animal models of HTN. These immune cells produce cytokines (e.g., Interleukin-17A, IL-17A), ROS, and matrix metalloproteinases that modulate renal and vascular function. IL-17A activates NADPH oxidase to stimulate the production of vascular superoxide, which in turn inhibits eNOS, leading to reduced NO bioavailability and a subsequent rise in blood pressure. Immune activation and resultant HTN-related organ dysfunction act synergistically to amplify blood pressure elevation and promote end-organ damage in HTN [74].

### 4.7. Gut Microbiome

Dysbiosis, a condition characterized by an altered gut microbiome, is also associated with HTN. Sodium, as a dietary component, can influence fluid homeostasis and immune system activation, but it can also modulate blood pressure and promote HTN by modifying the gut microbiome [75,76].

## 5. Current Standard Therapies, Preventive Approaches, and Curative Approaches for AS and HTN

### 5.1. Current Standard Therapies

Lowering LDL-C and apolipoprotein B (ApoB)-containing lipoproteins is one of the most effective strategies to reduce the risk of atherosclerotic cardiovascular disease (ASCVD) [77]. Statins are the mainstay of AS management due to their ability to reduce major adverse cardiovascular events, stabilize plaque, and lower LDL-C [78]. Ezetimibe and proprotein convertase subtilisin/Kexin Type 9 (PCSK9) inhibitors (Alirocumab, Evolocumab) significantly reduce LDL levels [79]. Bempedoic acid, which is particularly beneficial for people who are intolerant to statins and Inclisiran, a twice-yearly siRNA therapy that targets PCSK9 production, broaden the therapeutic landscape [79,80]. The first-line medication classes—ACE inhibitors, angiotensin II receptor blockers (ARBs), dihydropyridine calcium channel blockers, and thiazide/thiazide-like diuretics—continue to be the mainstay of pharmacological management of HTN [78].

### 5.2. Preventive Therapeutic Approaches

Reducing long-term exposure to atherogenic lipoproteins is the main strategy for preventing AS [81]. Along with pharmacological approaches, implementing lifestyle strategies such as the Mediterranean diet, weight control, smoking cessation, and physical activity factors lowers AS risk [82]. Recent preventive strategies such as coronary artery calcium (CAC) scoring, ApoB measurement, Lp(a) testing, and genetic screening for familial hypercholesterolemia emphasize the early identification of subclinical AS based on advanced risk stratification [83]. PCSK9 inhibitors and sodium-glucose co-transporter 2 (SGLT2) inhibitors are emerging therapies increasingly added to preventive strategies to reduce vascular and cardiometabolic risk [84].

Addressing environmental and lifestyle risks is the main goal of HTN prevention. The Dietary Approaches to Stop HTN (DASH) diet, a sodium intake below 1,500 mg/day, and the use of potassium-based salt substitutes have been shown to lower BP [85]. Risk reduction right from childhood and adolescence by restricting ultra-processed food intake, preventing early obesity, and adopting regular physical activity are being incorporated into preventive approaches for HTN [86]. Smoking cessation, weight loss, and potassium intake (≥3.5 g/day) remain essential preventive strategies for HTN [87].

### 5.3. Curative Approaches

Modern interventions can alter disease biology, stabilize plaque, and restore perfusion, leading to significant reductions in cardiovascular morbidity and mortality [88]. The term “curative” in modern cardiovascular science refers to prognostically transformational, disease-modifying treatments that change the course of HTN and ASCVD rather than simply managing symptoms [89].

Although AS cannot be completely reversed, modern therapies can significantly reduce inflammation, stabilize vulnerable lesions, and reduce plaque burden [90]. High-intensity statins enhance fibrous cap integrity and reduce lipid core volume, both of which are key features of plaque regression [91]. PCSK9 inhibitors have demonstrated signs of plaque regression and have significantly reduced LDL-C levels [90]. Inclisiran and bempedoic acid are potential disease-modifying agents that can reduce LDL-C over time in individuals intolerant to statin therapy [80].

Primary aldosteronism is one of the most prevalent reversible secondary causes of HTN and is targeted by treatments such as mineralocorticoid receptor antagonists or adrenal surgery [92]. Renal denervation is a procedure-based treatment option for treatment-resistant HTN that has demonstrated long-lasting reductions in BP [93,94].

## 6. Nutraceuticals—An Umbrella Term

Rising treatment costs associated with chronic human diseases impact the socioeconomic status of the population [9]. Therefore, there is a growing interest in safer, nutrition-based emerging alternative approaches known as nutraceuticals [10]. The term *nutraceutical* was first coined by Dr. Stephen De Felice in 1989, combining the words “Nutrition” and “Pharmaceutical,” suggesting that nutraceuticals bridge the gap between food and medicine. Mounting preclinical and clinical scientific evidence supports the potential health benefits of nutraceuticals beyond basic nutritive value. Nutraceuticals have received considerable attention because of their presumed safety and potential nutritional and therapeutic effects. In addition, it is suggested that nutraceuticals delay the aging process, increase life expectancy, and support the structure or function of the body [11]. A nutraceutical is defined as *a food or part of a food that provides medical or health benefits*, including the prevention and treatment of disease [95]. The term “nutraceutical” lacks a universal definition, and different organizations and nations may utilize different terminology or emphasize different aspects under their regulatory frameworks and guidelines [96]. Nutraceuticals are available in different forms such as powders, tablets, capsules, and beverages. They often contain isolated nutrients (e.g., vitamins, minerals, amino acids), herbal extracts, dietary supplements, genetically engineered designer foods, and fortified foods (cereals, soups, and beverages) [9,97,98].

The definition of nutraceuticals has evolved significantly over the past few years. Although nutraceuticals are also known as dietary supplements or functional foods, they differ in form, regulation, and how they are consumed [99]. The term nutraceutical is an umbrella term that includes both dietary supplements and functional foods, but each category has its own niche [10,99,100]. Despite having different definitions, legal contexts, and intended applications, the phrases nutraceuticals, dietary supplements, and functional foods are sometimes used interchangeably [101]. Nutraceuticals are products derived from food sources that offer health benefits beyond nutrition, and they can be taken as a food or a supplement. Dietary supplements are defined as *products intended to supplement the diet and are taken separately from meals* [102]. These are available in the form of pills, tablets, powders, soft gels, and liquids (e.g., multivitamins, fish oil, folic acid, calcium, and zinc tablets). Functional foods are *foods that are consumed as part of the daily diet* and are available in the form of whole or processed foods (e.g., probiotic yogurt, vitamin D-fortified milk, cereals, and juices) [101].

Nutraceuticals can be classified in several ways based on different criteria such as their natural source of origin, chemical nature, traditional use, therapeutic function, regulatory form, or their food category. Based on the existing literature [11,98,100,101,103,104], different classification systems for nutraceuticals and representative examples are summarized below.

Based on source of origin, nutraceuticals can be classified into plant-based (e.g., curcumin, resveratrol, flavonoids, polyphenols), animal-based (e.g., omega-3 fatty acids from fish oil, collagen, gelatin, and conjugated linoleic acid), and microbial-based (e.g., probiotics and fermented products like yoghurt, sauerkraut, soy products, tempeh, kefir, kimchi, kombucha). Based on the presence of a particular chemical group, they are categorized into alkaloids (e.g., caffeine, piperine, berberine, capsaicin), vitamins (e.g., vitamin D and multivitamins), minerals (e.g., zinc, calcium, and magnesium), polyunsaturated fatty acids (PUFAs) (e.g., omega-3, omega-6 fatty acids), polyphenols (flavonoids, catechins, anthocyanins), terpenoids (e.g., carotenoids), dietary fibers (e.g., celluloses, hemicelluloses, gums and pectins, lignin, beta glucans, and inulin), probiotics—live beneficial bacteria (e.g., Lactobacilli species, some cocci such as *Lactococcus lactis, Enterococcus faecium*, and some bifidobacteria species), prebiotics (the fibers that feed probiotics) (e.g., fructose-based oligosaccharides, raffinose and stachyose), and bioactive peptides (e.g., collagen and soy peptides).

Nutraceuticals can also be categorized based on their primary health benefits into different types such as antioxidant (polyphenols, vitamins E and C), anti-inflammatory (curcumin and resveratrol), anti-diabetic (e.g., fenugreek, chromium, berberine), anti-cancer, cardioprotective (e.g., omega-3), neuroprotective (e.g., *Ginkgo biloba*, phosphatidylserine), immunomodulatory (e.g., zinc, echinacea, probiotics).

Another system classifies nutraceuticals into conventional (or natural or traditional) nutraceuticals and non-conventional (or non-traditional/artificial/modified) nutraceuticals [105,106]. While the former category is natural food-based products with no manual alteration, the non-traditional category comprises products that are altered through fortification (fortified foods), formulation (designer foods), or genetic engineering (recombinant nutraceuticals).

Undoubtedly, there is growing interest in screening, evaluating, and validating several nutraceuticals pre-clinically and clinically. The subsequent Section 6 and Section 7 of this review discuss mechanistic insights into nutraceutical-mediated modulation of pathophysiological mechanisms involved in AS and HTN, drawing from recent in vitro and in vivo studies conducted in the last decade (2016–2025).

## 7. Nutraceuticals in the Modulation of AS

### 7.1. Polyphenols

- Curcumin (CUR)

**Figure 2:**
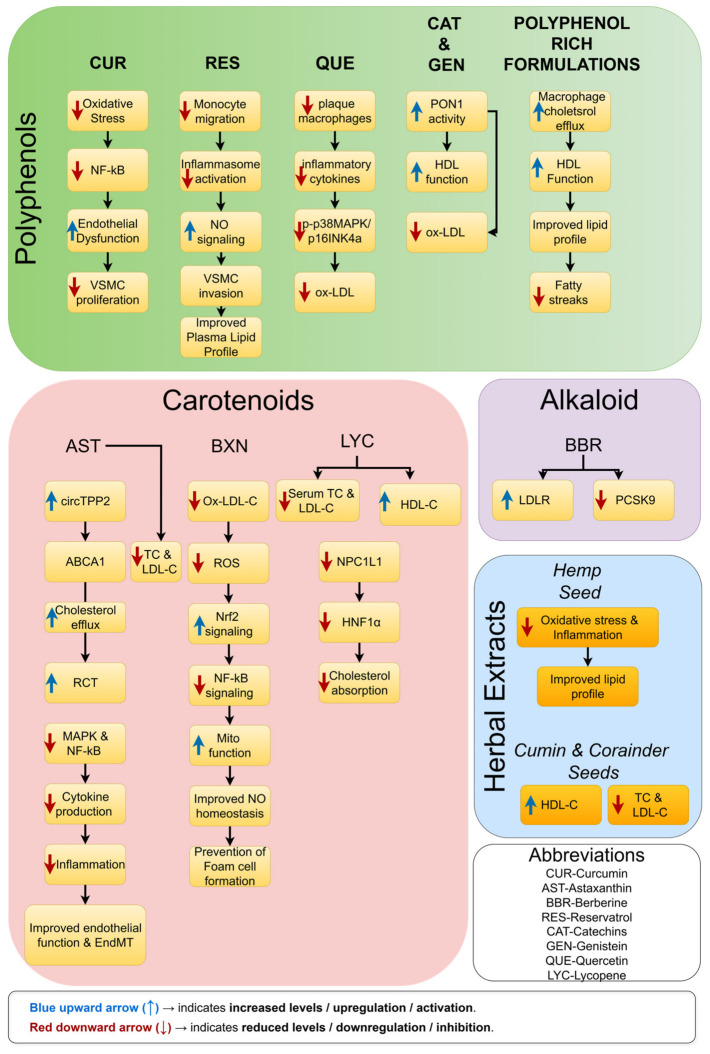
The underlying anti-atherosclerotic molecular mechanisms of polyphenols, carotenoids, alkaloids, and herbal extracts. Abbreviations: NF-κB: nuclear factor kappa-light-chain-enhancer of activated B cells; VSMC: vascular smooth muscle cell; NO: nitric oxide; ox-LDL: Oxidized Low-Density Lipoprotein; PON1: paraoxonase-1; HDL: high-density lipoprotein; ox-LDL-C: Oxidized Low-Density Lipoprotein Cholesterol; circTPP2: Circular RNA TPP2; ABCA1: ATP-Binding Cassette Transporter A1; RCT: reverse cholesterol transport; MAPK: Mitogen-Activated Protein Kinase; Nrf2: nuclear factor erythroid 2-related factor 2; Mito: mitochondria; TC: total cholesterol; LDL-C: low-density lipoprotein cholesterol; HDL-C: high-density lipoprotein cholesterol; NPC1L1: Niemann–Pick C1-Like 1; HNF1α: hepatocyte nuclear factor-1 alpha; EndMT: endothelial-to-mesenchymal transition; PCSK9: proprotein convertase subtilisin/kexin type 9; LDLR: Low-Density Lipoprotein Receptor.

Curcumin has been demonstrated to exhibit broad anti-atherogenic activity by targeting oxidative stress, inflammatory signaling (e.g., NF-κB), endothelial dysfunction, and vascular smooth muscle cell proliferation (Figure 2). Preclinical and clinical studies support its role in attenuating AS and related cardiovascular pathologies. However, a key translational challenge associated with curcumin is its low bioavailability [107].

- Resveratrol (RES)

Resveratrol exhibits anti-atherosclerotic effects by regulating energy metabolism, stress resistance, nitric oxide signaling, and gut microbiota composition [108]. Recent in vitro and in vivo studies by Alahmadi et al. (2026) [109] demonstrated that resveratrol’s anti-atherogenic effects may be attributed to its antioxidant and anti-inflammatory properties. Resveratrol inhibited ROS production, inflammasome activation, chemokine-induced monocyte migration, MMP activation, pro-inflammatory gene expression, and smooth muscle cell invasion in vitro. Resveratrol supplementation improved plasma lipid profile in high-fat diet-fed LDLR-deficient mice (Figure 2). Additionally, LDLR-deficient mice showed a reduction in macrophages and T-cell infiltration, along with increased collagen content in atherosclerotic plaques. Although preclinical findings suggest its cardiovascular protective potential, clinical evidence remains heterogeneous and limited.

- Quercetin (QUE)

Apolipoprotein E (ApoE) is essential for the clearance of circulating lipoproteins. Thus, deficiency of this gene results in hypercholesterolemia and AS. In vivo studies demonstrated that QUE exerts its anti-atherosclerotic effects by suppressing the accumulation of senescent plaque macrophages and secretion of senescence-associated inflammatory cytokines in high-fat diet-fed ApoE^−/−^ mice. Additionally, QUE supplementation significantly suppressed phosphorylation of p38MAPK and expression of p16INK4a, a cellular senescence marker, thereby attenuating AS in mice. In vitro, quercetin treatment substantially reversed oxLDL-induced senescence in macrophage cells. Overall, these findings suggest that quercetin’s anti-atherosclerotic effects are mediated through suppression of inflammation via inhibition of the p38MAPK/p16 pathway (Figure 2) [110].

It is important to note that flavonoid-rich nutraceuticals, including catechins (CAT) and genistein (GEN), enhance the activity of paraoxonase-1 (PON1). Increased PON1 activity improves HDL functionality and reduces LDL oxidation, thereby mitigating key processes in plaque formation (Figure 2) [111].

Oleactiv^®^, a polyphenol-rich formulation derived from artichoke, olive, and grape extracts, has been reported to significantly reduce aortic fatty streak formation and improve lipid profiles in hypercholesterolemic hamsters. Importantly, Oleactiv^®^ exerts its anti-atherosclerotic effects by enhancing macrophage cholesterol efflux (Figure 2), suggesting improved HDL functionality and reverse cholesterol transport (RCT) [112].

### 7.2. Carotenoids

- Astaxanthin (AST)

Reverse cholesterol transport (RCT) is an important anti-atherogenic mechanism by which HDL removes excess cholesterol from peripheral arterial walls and from foam cells (LDL-C-rich macrophages) within atherosclerotic plaques and transports it back to the liver for excretion in bile. In vitro studies revealed that cholesterol efflux from foam cells is regulated by the circTPP2/miR-3073b-5p/ATP-binding cassette subfamily A member 1 ABCA1 axis [113]. Astaxanthin (AST) is a xanthophyll carotenoid with a myriad of health benefits and has emerged as a promising nutraceutical for the management of CVDs. AST treatment has been shown to significantly increase the expression of circTPP2, which leads to increased cholesterol efflux from foam cells via modulation of ABCA1 [113]. In vivo studies also showed that astaxanthin increases RCT in mouse models of AS [114]. Recent studies have shown that astaxanthin treatment significantly attenuates inflammation by suppressing the MAPK pathway and reduces atherosclerotic lesion size in LDLR-deficient mice [115]. Anti-inflammatory effects of astaxanthin have been attributed to its ability to reduce cytokine production and enhance NO bioavailability through the regulation of NF-κB and MAPK signaling pathways (Figure 2). Such downregulation of inflammatory signaling is essential for the improvement of endothelial function and may also help prevent endothelial–mesenchymal transition, a key process contributing to the development of atherosclerotic plaques [116]. Previous clinical trials showed that astaxanthin supplementation significantly reduced both total cholesterol (TC) and LDL-C levels, but had no effect on TG and HDL-C levels, in patients with coronary artery disease, indicating astaxanthin’s potential anti-atherogenic effects [117].

- Bixin (BXN)

In vitro studies showed that bixin pretreatment reduced oxLDL-induced atherogenic signaling in macrophages through prevention of ROS generation, restoration of NO homeostasis, attenuation of mitochondrial dysfunction, and inhibition of foam cell formation. Furthermore, bixin treatment upregulated the nuclear factor erythroid 2-related factor 2 (Nrf2) signaling pathway and downregulated the NF-kB pathway (Figure 2). These findings further suggest that bixin may exert greater anti-atherosclerotic potential than lycopene, a well-known nutraceutical compound used as a positive control in this study [118].

- Lycopene (LYC)

Lycopene, primarily found in tomatoes, has been widely used as a dietary supplement due to its diverse biological activities. Lycopene supplementation has been shown to significantly reduce serum TC and LDL-C levels while increasing HDL-C levels (Figure 2). Lycopene was also shown to decrease the expression of Niemann–Pick C1-Like 1 (NPC1L1), a key intestinal cholesterol transporter, and its transcriptional regulator, hepatocyte nuclear factor-1α (HNF-1α), in the small intestine, thereby reducing intestinal cholesterol absorption (Figure 2) and attenuating the progression of AS in ApoE^−/−^ mice fed a high-fat diet (HFD) [119].

### 7.3. Alkaloid Berberine (BBR)

Mutations in genes including LDLR, apolipoprotein B (APOB), and proprotein convertase subtilisin/kexin type 9 (PCSK9) lead to the genetic disease familial hypercholesterolemia (FH). It is characterized by the inability to clear LDL-C from the blood, resulting in the accumulation of elevated levels of bad cholesterol, consequently increasing the risk of developing AS [120] and other CVDs such as stroke and heart attack. The LDLR plays a crucial role in the clearance of blood LDL-C. LDLR recognizes and binds to apolipoprotein B100 (ApoB100) present on the LDL particles and to apolipoprotein E on the remnant lipoproteins (chylomicron remnants, VLDL remnants, and IDL). Additionally, proprotein convertase subtilisin/kexin type 9 (PCSK9) is critical for controlling LDL-C levels in the blood. Therefore, induction of LDLR or inhibition of PCSK9 by natural compounds or nutraceuticals represents a promising strategy for reducing the burden of CVDs [121]. Berberine, an alkaloid, has been reported to function as a hepatic LDLR inducer and to inhibit the expression of the PCSK9 protein (Figure 2) [122].

A recent development in targeting nutraceuticals is the use of plant-derived extracellular vesicles as carriers for the delivery of nutraceuticals, particularly those with low bioavailability and stability. Ginkgetin plus berberine-loaded extracellular vesicles derived from avocado significantly decreased the expression of both NFκB and NOD-like receptor family pyrin domain–containing 3 (NLRP3). The expression of pro-inflammatory and atherogenic genes, including Cd36, Tnfα, Il1β and Il6, was suppressed, and formation of macrophage foam cells was also attenuated by treatment with bioactive-loaded extracellular vesicles. These findings suggest the superior anti-atherogenic effects of plant-derived EV-loaded nutraceuticals [123].

### 7.4. Medicinal Herbal Extracts

Hemp seed (*Cannabis sativa*) extract is rich in omega–3 and omega–6 PUFA, suggesting its ability to modulate oxidative stress and inflammation (Figure 2). Recent studies reported that hempseed extract significantly ameliorated high-fat diet–induced increases in oxidative stress, inflammation, and altered lipid profile in vivo, suggesting anti-hypercholesterolemic effects [124]. Inflammation is known to impair reverse cholesterol transport (RCT) by reducing the expression of two important cholesterol efflux transporters, ABCA1 and ABCG1. The omega-3 PUFA of hemp seeds may prevent inflammation-induced downregulation of efflux transporters, thereby protecting cholesterol efflux from peripheral macrophages and boosting RCT efficiency, thus exerting anti-inflammatory and anti-atherosclerotic benefits.

Cumin and coriander seeds, which are widely used for culinary purposes, are well-known flavoring agents, and recent in vivo studies suggest their nutraceutical benefits against AS–related CVDs. Both cumin- and coriander-seed-mixed diets were found to enhance HDL-C levels in rats fed a fat-rich diet for two weeks (Figure 2). Notably, cumin supplementation alone reduced total lipid and LDL-C levels and lowered abdominal fat accumulation [125].

### 7.5. Beta-Glucan, a Soluble Dietary Fiber

Beta-glucans, a class of dietary fiber-based nutraceuticals, attenuate AS through cholesterol- and glucose-lowering effects, antioxidant activity, immunomodulation, and gut microbiota regulation [126]. Accumulating evidence suggests a positive correlation between the consumption of soluble dietary fibers and reduced risk of CVDs. Dietary fibers such as β-glucan, pectin, guar gum, and psyllium have been demonstrated to lower serum cholesterol levels. The cholesterol-lowering effects of dietary fibers occur mainly through three key mechanisms. First, by binding to bile salts, thereby preventing their reabsorption from the small intestine, resulting in the excretion of high amounts of bile salts in feces. The second mechanism by which dietary fibers reduce cholesterol levels is by stimulating low hepatic cholesterol synthesis. The third mechanism involves inhibition of cholesterol synthesis by propionate, a fermentation product of dietary fibers [127]. Beta-glucan, a natural polysaccharide present in the cell walls of oats, barley, and wheat, has been reported to possess nutraceutical benefits [126]. Beta-glucan has been reported to demonstrate free radical scavenging activity, reducing blood cholesterol and glucose levels, thereby lowering the risk of AS [128,129]. Additionally, β-glucan from barley has been found to lower TC by interrupting bile acid metabolism [130].

### 7.6. Phytochemical-Based Nutraceutical Formulations

Statins are drugs prescribed to prevent heart attacks and strokes by lowering LDL-C through the inhibition of cholesterol-producing enzymes in the liver. Ezetimibe is a cholesterol-absorption inhibitor that reduces LDL-C by blocking the absorption of cholesterol in the small intestine. A nutraceutical formulation composed of ezetimibe (EZE) and a nutraceutical compound (NC, containing monacolin-K, Berberine Hydrochloride, resveratrol, quercetin, and chromium) has been evaluated for its effectiveness and safety on lipid profiles in statin-intolerant patients with moderate-to-high CV risk. Three-month supplementation with EZE + NC caused a significant reduction in LDL-C and TC and increased HDL-C levels in statin-intolerant patients compared to patients who received only EZE [131].

Several other plant-based nutraceutical formulations have been shown to remarkably modulate lipid profiles in clinical trials involving subjects with hypercholesterolemic patients [132,133,134,135]. Armolipid Plus^®^ and LopiGLIK^®^ are also phytochemical-based nutraceuticals. Armolipid Plus^®^ is composed of natural ingredients such as Berberine, Red Yeast Rice, Policosanol, Folic Acid, Coenzyme Q10, and Astaxanthin, while LopiGLIK^®^ is contains berberine, red yeast rice powder, and *Morus alba*. Clinically, it was shown that supplementation with Armolipid Plus^®^ and LopiGLIK^®^ significantly reduced plasma levels of TC, LDL-C, and TG. Additionally, both treatments lowered systolic and diastolic blood pressure. However, the effects of LopiGLIK^®^ on lipid profile and blood pressure were found to be superior to those of Armolipid Plus^®^ [132].

Eufortyn^®^ Colesterolo Plus is a nutraceutical formulation composed of standardized bergamot polyphenolic fraction phytosome, artichoke extract, Q10 phytosome and zinc. In a clinical trial, eight–week supplementation with Eufortyn^®^ Colesterolo Plus led to a significant reduction in TC, LDL-C, non-HDL-C, high-sensitivity C-reactive protein (hs-CRP) and endothelial reactivity in subjects with moderate hypercholesterolemia compared to placebo treatment [133]. Likewise, supplementation with a nutraceutical formulation, Zeta Colest, composed of *Berberis aristata* dry extract, berberine, red yeast rice extract, milk thistle dry extract, guggul dry extract, exerted significant lipid-lowering effects in mildly hypercholesterolemic patients [134].

### 7.7. Red Yeast Rice

Red yeast rice (RYR) is a fermented nutraceutical prepared by fermenting ordinary rice with *Monascus purpureus*, a fungus. It is widely used as a nutraceutical due to its lipid-lowering effects. RYR has been reported to reduce cholesterol levels in the body [136]. The cholesterol-lowering effects of RYR are attributed to the presence of monacolins (e.g., monacolin K), important bioactive compounds, which are important bioactive compounds that act by inhibiting enzymes of cholesterol biosynthesis, particularly hydroxymethylglutaryl-coenzyme A (HMG-CoA) reductase [137].

Overall, nutraceuticals modulate AS through complementary, multi-level mechanisms involving oxidative stress reduction, inflammation resolution, endothelial protection, immune modulation, and cholesterol trafficking. Their integration into preventive or adjunct therapeutic strategies offers a rational approach to addressing LDL-independent residual cardiovascular risk [138,139,140]. A comparative summary of mechanistic, preclinical, and clinical evidence for nutraceuticals with anti-atherogenic potential is provided in Table 1. Although a number of compounds exhibit promising biological effects, clinical validation remains limited and inconsistent.

## 8. Nutraceuticals in the Modulation of HTN

### 8.1. Antioxidant Phytochemicals

**Figure 3:**
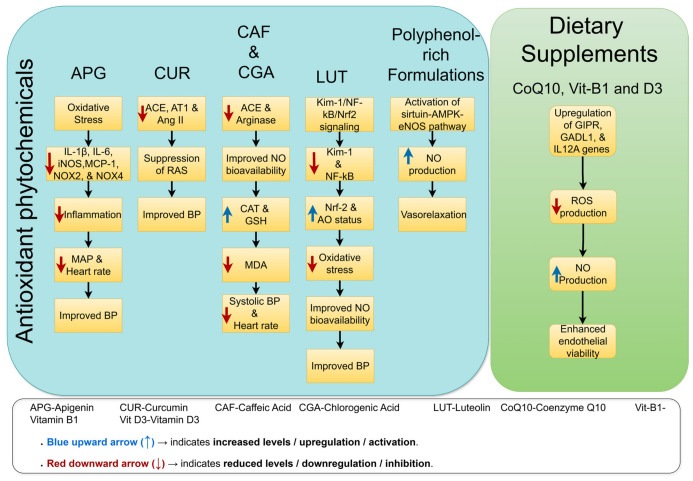
Antihypertensive molecular mechanisms of antioxidant phytochemicals and dietary supplements. Abbreviations: IL-1β: Interleukin-1 Beta; IL-6: Interleukin-6; iNOS: Inducible Nitric Oxide Synthase; MCP-1: Monocyte Chemoattractant Protein-1; NOX2: NADPH oxidase 2; NOX4: NADPH oxidase 4; MAP: mean arterial pressure; BP: blood pressure; ACE: angiotensin-converting enzyme; AT1: angiotensin receptor type 1; Ang II: angiotensin II; RAS: renin–angiotensin system; NO: nitric oxide; Nrf2: nuclear factor erythroid 2-related factor 2; CAT: catalase; GSH: glutathione; Kim-1: Kidney Injury Molecule-1; NF-κB: nuclear factor kappa-light-chain-enhancer of activated B cells; AO: antioxidant; AMPK: AMP-Activated Protein Kinase; eNOS: endothelial nitric oxide synthase; ROS: reactive oxygen species; GIPR: Gastric Inhibitory Polypeptide Receptor; GADL1: Glutamate Decarboxylase-Like Protein 1; IL12A: Interleukin-12 Subunit Alpha.

Apigenin has been reported to reduce oxidative stress and inflammation, thereby improving blood pressure and heart enlargement in spontaneously hypertensive rat (SHR) models. Particularly, apigenin infusion into the periventricular nucleus (PVN) of the hypothalamus resulted in a reduction in mean arterial pressure (MAP), heart rate, circulating norepinephrine (NE), and oxidative stress in both the heart and the PVN. Additionally, the levels of inflammatory markers such as IL-1β, IL-6, iNOS, monocyte chemotactic protein-1 (MCP-1), NOX2, and NOX4 were all reduced (Figure 3) [145].

Curcumin’s myriad biological effects, including antioxidant, anti-inflammatory, antihypertensive and lipid-lowering effects, have been reported in several clinical studies, indicating curcumin’s potential in modulating several risk factors of CVDs [146]. RAS in the brain can regulate both cognitive functions and BP. Therefore, inhibition of RAS by drugs may be a good strategy for the improvement of cognitive function in hypertensive patients. To this extent, Lim et al. investigated whether curcumin supplementation can affect the cognitive function and hypertensive–related markers in scopolamine-treated spontaneously hypertensive rat/Izm (SHR/Izm). Their results showed that curcumin (100 mg and 200 mg) reduced the mRNA levels of both ACE and angiotensin II receptor type1 (AT1), and reduced Ang II levels in the brain. Additionally, a significant increase in the mRNA levels of the muscarinic acetylcholine receptors (mAChRs) and subsequent increase in the acetylcholine content were observed, thereby improving BP and cognitive function in SCO-induced hypertensive mice. These findings indicate that curcumin enhances the cholinergic system by suppressing RAS and AT1 receptor expression (Figure 3) while increasing mAChR expression [147].

Polyphenols such as caffeic acid and chlorogenic acid have been shown to significantly reduce systolic blood pressure, heart rate, and the activity of ACE and arginase in cyclosporine-induced hypertensive rats (Figure 3). Additionally, these two polyphenols improved NO bioavailability, increased catalase activity and glutathione (GSH) content, while reducing MDA levels (Figure 3). These findings suggest that caffeic acid and chlorogenic acid exert blood pressure-lowering potential by reducing the activities of key enzymes linked to the pathogenesis of HTN in cyclosporine-induced rats [148]. Nω-Nitro-L-arginine-methylester (L-NAME) is a synthetic analog of L-arginine, which is used as a non-selective inhibitor of NOS in experimental systems of HTN. L-NAME treatment reduces NO production, thereby impairing vasodilation in experimental rats. The combination of caffeine, an alkaloid and caffeic acid, a polyphenol, significantly improved systolic blood pressure, possibly by reducing the activities of ACE and arginase enzymes as well as the levels of MDA, while increasing NO levels in L-NAME-induced hypertensive rats [149].

Oyagbemi et al., 2018 [150] showed in vivo that administration of luteolin, a plant-based polyphenolic flavonoid, prevented sodium fluoride-induced significant rise in both systolic and diastolic BP, mean arterial pressure, and a substantial increase in oxidative stress markers (e.g., MDA, PCCs, MPO) and prevented decreases in enzymatic and non-enzymatic antioxidant levels (e.g., SOD, CAT, GR, GPx, GSH) and reduced NO bioavailability. Additionally, luteolin treatment prevented sodium fluoride-induced upregulation of kidney injury marker 1 (Kim-1), NF-κB, Nrf2, and cardiac troponin I (cTnI). These findings suggest that luteolin exerts its antihypertensive and cardiovascular protective effects primarily through modulation of Kim-1/NF-kB/Nrf2 signaling pathways (Figure 3) [150].

Taurisolo^®^ is a nutraceutical product composed of a polyphenol-rich (catechin and procyanidins) extract from the Aglianico cultivar grape. In vitro studies revealed that Taurisolo^®^ activates the sirtuin–AMPK-eNOS pathway leading to NO production and subsequent promotion of vasorelaxation (Figure 3). Moreover, Taurisolo^®^ inhibited cardiac hypertrophy, hypercoagulability and HTN and improved endothelial function [151].

### 8.2. Dietary Supplements

A recent study investigated the beneficial effects of nutraceuticals such as coenzyme Q10, vitamin B1, and vitamin D3 on aortic valve endothelial cells. The results showed that these nutraceuticals reduced ROS production, enhanced endothelial viability, and increased NO production (Figure 3). Beneficial effects were reflected in the upregulation of anti-atherosclerotic gene GIPR, antioxidant genes GADL1 and CPM, and anti-inflammatory gene IL12A and CPM (Figure 3). Furthermore, daily oral intake of a combination of these bioactives for four weeks improved endothelial function in participants with pre-existing endothelial dysfunction and reduced arterial stiffness without evidence of hepatic toxicity [152].

### 8.3. Bioactive Peptides

**Figure 4:**
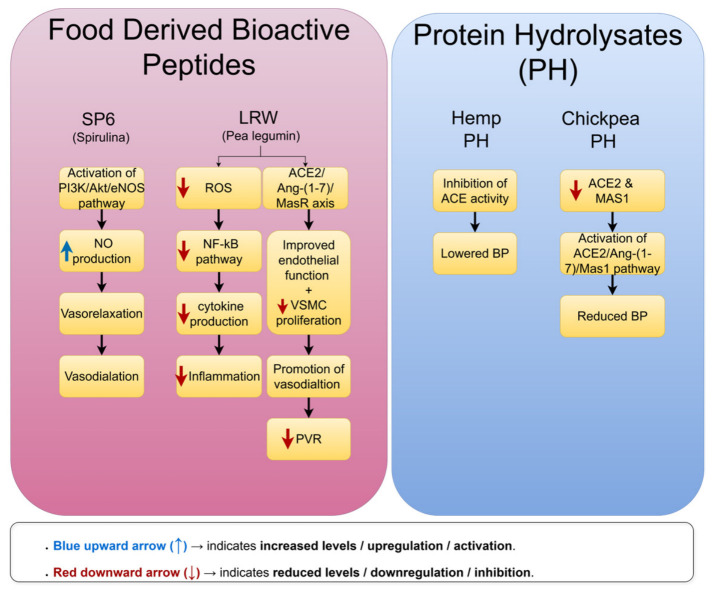
Antihypertensive molecular mechanisms of bioactive peptides and protein hydrolysates. Abbreviations: PI3K/Akt/eNOS pathway: phosphoinositide 3-kinase/Protein Kinase B/endothelial nitric oxide synthase pathway; NO: nitric oxide; ROS: reactive oxygen species; NF-κB: nuclear factor kappa-light-chain-enhancer of activated B cells; VSMC: vascular smooth muscle cell; ACE2/Ang(1-7)/MasR axis: angiotensin-converting enzyme 2/angiotensin(1-7)/Mas Receptor axis; PVR: peripheral vascular resistance; MAS1: Mas Receptor 1; ACE: angiotensin-converting enzyme; BP: blood pressure.

Several food-derived peptides have been shown to exhibit a promising nutraceutical strategy for the management of HTN. Cú-Cañetas et al., 2026, showed that peptide fractions prepared from cowpea (*Vigna unguiculata*) seed proteins exhibit a non-competitive ACE-I inhibitory potential in vitro [153]. Wang et al., 2020 [154], isolated a tripeptide, LRW (Leu-Arg-Trp), from pea protein legumin. LRW treatment decreased angiotensin II-induced superoxide production, inflammation, and proliferation in VSMCs. LRW upregulated the ACE2-Ang-(1-7)-MasR axis and modulated the nuclear factor-κB (NF-kB) pathway. The ACE2-Ang-(1-7)-MasR axis promotes vasodilation, reduces vascular resistance, and improves endothelial function (Figure 4). Moreover, this axis can reduce NF-kB activation and cytokine production, thereby exerting anti-inflammatory effects (Figure 4). These findings suggest that LRW may serve as a functional food ingredient or nutraceutical for the prevention of vascular damage and management of HTN [154]. Other studies also demonstrated the ACE-I inhibitory effects of chickpea (*Cicer arietinum* L.) peptides [155].

Carrizzo et al. (2019) [156] isolated a decapeptide, SP6 (GIVAGDVTPI) from spirulina. Their ex vivo study results reported that SP6 exerts direct endothelium-dependent vasodilation via activation of the phosphoinositide-3-kinase/serine/threonine kinase (PI3K/Akt) pathway, which promotes the production of NO. Administration of SP6 induced a significant hemodynamic effect, reducing BP in eNOS-deficient mice. Of note, although lower doses of SP6 had no hemodynamic effects, they still enhanced endothelial NO-mediated vasorelaxation. Additionally, SP6 was shown to exert an antihypertensive effect by improving endothelial vasorelaxation in an experimental model of arterial HTN. Altogether, these findings suggest that SP6 reduces BP through modulation of the PI3K/AKT/eNOS pathway (Figure 4) [156].

### 8.4. Protein Hydrolysates

Several of the plant- and animal-based protein hydrolysates have been demonstrated to exhibit antihypertensive potential in vitro and in vivo models. Gum–based nutraceutical formulations of hydrolyzed plant materials have gained significant interest due to enhanced functional properties and bioavailability, prebiotic and gut health-promoting properties. Incorporation of hemp protein hydrolysate in pectin-based gummy candies resulted in higher levels of polyphenol content, consequently significant antioxidant activity. A gummy candy enriched with hydrolyzed hemp (*Cannabis sativa* L.) was shown to enhance the ACE inhibitory activity in vitro (Figure 4), indicating hydrolyzed hemp as a stable ingredient for the preparation of plant-based nutraceutical formulations targeting oxidative stress and HTN [157]. Similarly, chickpea protein hydrolysates have also been shown to exhibit antihypertensive potential in vivo in SHRs. Oral supplementation of chickpea protein hydrolysates for five weeks led to a significant increase in the mRNA expression of ACE2 and Mas1 levels in the kidneys. But mRNA levels of angiotensin-converting enzyme-I (ACE1), renin, and AT_1_R receptor remained the same in treated and control groups. Systolic, diastolic, and mean BP levels were also reduced. These results suggest that chick pea protein hydrolysates improve BP via activation of the ACE2/Ang-(1-7)/Mas1 pathway of the renin–angiotensin–aldosterone system (Figure 4) [158].

### 8.5. Herbal Nutraceuticals

- *Hibiscus sabdariffa*

**Figure 5:**
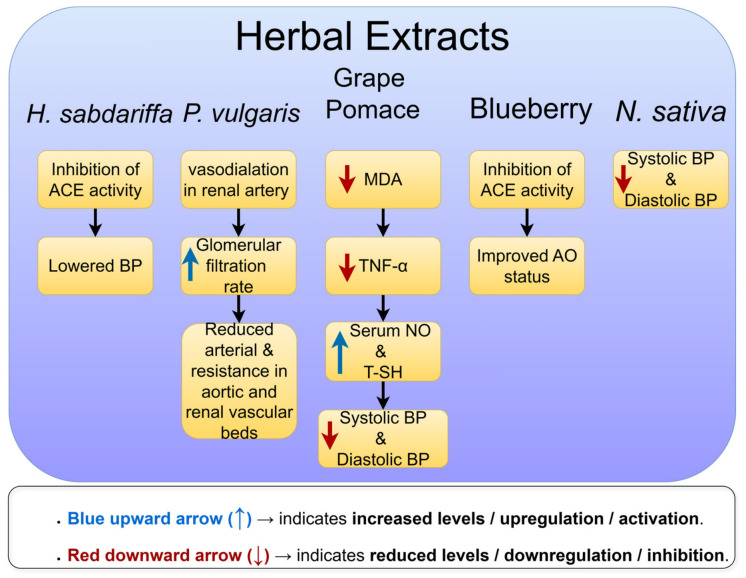
Antihypertensive molecular mechanisms of herbal extracts. Abbreviations: *H. sabdariffa*: *Hibiscus sabdariffa*; *P. vulgaris*: *Phaseolus vulgaris*; *N. sativa*: *Nigella sativa*; ACE: angiotensin-converting enzyme; MDA: Malondialdehyde; TNF-α: Tumor Necrosis Factor-Alpha; NO: nitric oxide; T-SH: Total Sulfhydryl Groups; BP: blood pressure.

Recent studies have highlighted the antihypertensive potential of H. sabdariffa, which is rich in phenolic acids and essential amino acids. Aqueous extract of calyx from *H. sabdariffa* demonstrated significant ACE-competitive inhibitory effects in vitro (Figure 5) and substantial diuretic and natriuretic effects in vivo, supporting its potential application in the development of plant-based nutraceutical formulations for the management of HTN [159].

- *Phaseolus vulgaris*

It has been reported that the protein extract (PV3) from *Phaseolus vulgaris* increases glomerular filtration rate in WT and hypertensive rats; causes natriuresis in SHR; promotes NO- and endothelium-dependent vasorelaxation in renal artery rings; reduces arterial pressure and resistance in aortic and renal vascular beds; and exerts antihypertensive effects (Figure 5) in a dose-dependent manner. These findings suggest that PV3 serves as a source of bioactive peptides and highlight its potential for inclusion in nutraceutical formulations aimed at treating renal dysfunction and HTN [160].

- Grape Pomace extract

Chronic administration of dexamethasone, a glucocorticoid, can increase oxidative stress and blood pressure in experimental rodent models. White grape pomace extracts remarkably prevented the accumulation of oxidative stress (MDA) and inflammatory markers (serum TNF-α, but not heart tissue TNF-α, IL-1β, and IL-6) (Figure 5) in experimental models of dexamethasone-induced hypertensive rats. GP extract also increased the serum levels of NO and total thiols, while significantly reducing systolic and diastolic blood pressure (Figure 5) [161].

- Blueberry

Freeze-dried blueberry powder is abundant in bioactive polyphenols such as quercetin, gallic acid, cyanidin chloride, vitamin C, trans-caffeic acid, procyanidin B1, and procyanidin B2. In vitro studies found that blueberry powder exhibits significant antioxidant activity and ACE-inhibitory activity (Figure 5) in a dose-dependent manner. This suggests possible antihypertensive effects of blueberry [162].

- *Nigella sativa*

Blood pressure is typically lower in premenopausal women than in men. However, after menopause, the prevalence of HTN in women exceeds that in men [163]. Recent pilot observational studies by Pala et al. (2025) [164] also reported that *Nigella sativa*, a medicinal herb, might be beneficial in the management of HTN. *N. sativa* supplementation was given to 52 postmenopausal women and resulted in a significant, dose-dependent reduction in seated office systolic and diastolic BP (Figure 5), supporting the role of *N. sativa* as an effective complementary therapy in HTN management in postmenopausal women [164].

A comparative summary of mechanistic, preclinical, and clinical evidence for nutraceuticals with antihypertensive potential is presented in Table 2. While several polyphenolic compounds (polyphenols such as curcumin, resveratrol, caffeic acid, chlorogenic acid) and bioactive peptides demonstrate promising antihypertensive and vascular protective effects [165,166,167,168], clinical validation remains inconsistent, underscoring the need for careful interpretation of results and the design of standardized clinical trials. On the other hand, omega-3 PUFAs constitute an important class of bioactive compounds that have been shown to exhibit clinically meaningful antihypertensive effects, as evidenced by several clinical trials and meta-analyses. Mechanistically, omega-3 PUFAs improve endothelial function and attenuate arterial inflammation, thereby reducing blood pressure, particularly diastolic blood pressure [169].

## 9. Summary of Clinical Evidence on Nutraceuticals in HTN and AS

HTN is one of the major causes of cardiovascular morbidity and mortality, with growing interest in adjunctive non-pharmacological strategies such as nutraceuticals [173]. Table 3 lists the clinical trials evaluating the effects of nutraceutical interventions, including antioxidant compounds, plant-derived extracts, amino acid derivatives, and dietary components across diverse populations and geographic regions [174,175,176]. These studies provide emerging evidence supporting the role of nutraceuticals in modulating cardiovascular risk factors, particularly HTN, endothelial function, inflammation, and other metabolic parameters.

Several randomized controlled trials demonstrated consistent findings showing a modest but significant reduction in blood pressure with some nutraceutical interventions. Combinations of red yeast rice with coenzyme Q10 demonstrated reductions in both systolic and diastolic blood pressure, alongside improvements in lipid and glucose profiles [177]. Similarly, plant-based and polyphenol-rich interventions, including tomato nutrient complexes, grape seed extract, and Sacha Inchi oil, were associated with reductions in blood pressure, often in a dose-dependent manner [178,179,180]. Notably, the tomato nutrient complex showed efficacy only at higher doses (≥15 mg lycopene), highlighting the importance of adequate bioavailability and synergistic interactions between bioactive compounds [179]. Likewise, beetroot dry extract combined with magnesium and vitamins D, B1 and C substantially reduced systolic and diastolic blood pressure in the medium term, leading to a significant reduction in estimated cardiovascular risk [181].

Beyond direct blood pressure-lowering effects, several nutraceuticals exerted beneficial vascular and metabolic effects through alternative mechanisms. For instance, L-citrulline supplementation improved endothelial function and vascular conductance [182], while cannabidiol reduced mean arterial pressure mediated via neurohormonal pathways including urotensin-II and catestatin [183,184]. Interventions such as green leafy vegetables and nitrate supplementation influenced the oral microbiome and nitric oxide pathways, further supporting the role of diet-derived compounds in vascular regulation [185].

The nitric oxide-soluble guanylate cyclase-cyclic guanosine monophosphate (sGC-cGMP) signaling pathway is a key mechanism for the synthesis of cGMP and normal functioning of the cardiovascular system in healthy individuals. A nutraceutical blend of grape pomace extract (Taurisolo^®^) rich in polyphenols and L-arginine, a natural substrate of endothelial NOS, was tested for its antihypertensive effects in individuals with grade 1 and grade 2 HTN. Findings of this study showed that the formulation improved the bioavailability of NO and activated sGC-cGMP signaling, thereby substantially reducing systolic and diastolic blood pressure, while renal function remained consistent during the study. This intervention was generally well accepted, with no significant negative effects in HTN patients, suggesting a promising and efficient nutraceutical approach to traditional antihypertensive treatment [186].

A prominent feature across studies is the antioxidant and anti-inflammatory potential of nutraceuticals. Compounds such as pycnogenol, alpha-lipoic acid, and coenzyme Q10 demonstrated reductions in oxidative stress markers and inflammatory mediators, along with improvements in endothelial function [187,188,189]. However, these biochemical and functional benefits did not always translate into significant blood pressure reductions, indicating that their primary role may lie in vascular protection and risk modification rather than direct antihypertensive effects.

The included studies also highlight the heterogeneity of responses based on population characteristics, baseline risk, and intervention type. For example, seaweed (*nori*) intake has been shown to lower blood pressure in children [190], suggesting potential early-life preventive benefits. Conversely, some interventions showed limited or no effect in specific subgroups or when baseline risk was lower [176]. Additionally, certain nutraceuticals may carry potential risks; for instance, alpha-lipoic acid was associated with alterations in iron metabolism, indicating the need for careful monitoring in clinical use [189].

In a single-center, open-label, case-control, pilot study, hypertensive patients receiving the nutraceutical mixture (trehalose, spermidine, nicotinamide, and polyphenols) for two months showed a significant improvement in arterial stiffness compared to hypertensive patients not receiving the mixture. This counteraction of HTN–related arterial stiffness was linked to reduced oxidative stress and autophagy stimulation, indicating that these autophagy activators may act as promising adjuvants for the prevention of HTN [191].

Previous clinical trials in young and middle-aged subjects who are supplemented with policosanol (10 mg/day) for eight weeks led to a significant reduction in serum TG and an increase in serum HDL-C levels, suggesting policosanol’s potent anti-atherosclerotic effects [192].

**Table 3:** Overview of clinical trials of nutraceuticals in HTN and AS.

Author and Year	Country	Study Population	Study Design	Study Drug/Comparator	Sample Size	Main Findings
Devaraj et al., 2007 [174]	United States of America	Patients with coronary artery disease	Randomized Controlled Trial	Intervention: RRR-α-tocopherol Comparator: Placebo	90	Reduced biomarkers of oxidative stress and inflammation but did not improve carotid AS or clinical cardiovascular outcomes
Kim et al., 2017 [192]	Republic of Korea	- Young non-smokers (YN): mean age 24.0 ± 2.4 years - Young smokers (YS): mean age 26.3 ± 1.5 years - Middle-aged subjects (MN): mean age 52.5 ± 9.8 years	Open-label clinical intervention study	Policosanol 10 mg/day (no placebo comparator; participants served as their own controls)	25	Significant reduction in systolic BP by ~7 mmHg (≈4%) in YS and MN groups Serum TG decreased 28% (YN) and 26% (MN), and HDL-C percentage increased (YN +36%, YS +35%, MN +8%). Reduced Cholesteryl Ester Transfer Protein (CETP) activity by 21% (YN) and 32% (MN). Paraoxonase (PON1) activity ↑ 17% ApoA-I and cholesterol content ↑ HDL particle size and number increased (2-fold in YS). Reduced LDL oxidation and apoB fragmentation.
Cicero et al., 2018 [181]	Italy	Pre-hypertensive and first-degree hypertensive patients	Pilot randomized, double-blind, placebo-controlled clinical trial	Intervention: BPLN^®^ nutraceutical composite (containing nitric oxide donor, magnesium, vitamins) Comparator: placebo	36	Significant reduction in morning BP parameters and evening systolic BP compared to baseline and placebo, with no side effects reported.
Carrizzo et al., 2020 [175]	Italy	Patients with uncontrolled HTN (age 40–68 years) on antihypertensive treatment	Randomized, double-blind clinical study (two parallel trials)	Intervention: AkP05 nutraceutical combination Comparators: Placebo and diuretic	69	Significant reduction in blood pressure Improved endothelial function and ↑ nitric oxide release Improved exercise capacity (↑ VO_2_ max, stress tolerance, power output) Mechanism: ↓ oxidative stress and ↑ NO Potential adjunct therapy for BP control
Ferguson et al., 2022 [176]	Australia	Healthy adults aged 55–75 years (some with normal-high SBP; some on/off BP medication)	Randomized, double-blind, placebo-controlled trial (secondary analysis)	Intervention: Polyphenol-rich supplement (*Pinus massoniana* bark extract, 1322 mg/day) Comparator: Placebo	62	Significant reduction in systolic BP (−3.29 mmHg, *p* = 0.028) at 12 weeks Greater reduction in normal-high SBP subgroup (−6.46 mmHg) Strongest effect in non-medicated individuals (↓ SBP and DBP) No significant effect on DBP overall No significant between-group differences
Kang et al., 2024 [182]	United States of America	Patients with Hypertensive postmenopausal women	Randomized, placebo-controlled trial	Intervention: L-citrulline (10 g/day for 4 weeks) Comparator: Placebo	22	Significant improvement in endothelial function (↑ FMD) Reduction in aortic systolic BP Improved exercise blood flow and vascular conductance Enhanced muscle oxygenation (↑ TSI, ↓ HHb) during exercise Benefits linked to improved endothelial function
Kumric et al., 2023 [183]	Croatia	Patients with mild to moderate essential HTN	Randomized crossover trial	Intervention: Cannabidiol (CBD) Comparator: Placebo	51	Significant reduction in serum urotensin-II levels after CBD Reduction in mean arterial pressure (MAP) correlated with decrease in urotensin-II No effect observed with placebo Suggests urotensin-II may mediate CBD-induced BP reduction
Lira et al., 2024 [178]	United States of America	Healthy males/individuals with elevated or stage 1 HTN	Randomized, double-blind, crossover trial	Intervention: Grape seed extract (GSE) Comparator: Placebo	10	Significant reduction in diastolic BP and mean arterial pressure No significant effect on systolic BP, heart rate, cardiac output, or HRV No change in autonomic (sympathetic) response BP reduction likely via peripheral vasodilation
Kumric et al., 2023 [184]	Croatia	Patients with Grade 1 HTN	Randomized, placebo-controlled, crossover trial	Intervention: Cannabidiol (CBD) Comparator: Placebo	54	Significant reduction in serum catestatin levels with CBD Reduction in mean arterial pressure (MAP) correlated with decrease in catestatin levels Baseline catestatin predicted BP response Suggests BP-lowering effect mediated via sympatho-chromaffin system
Ormesher et al., 2024 [193]	United Kingdom	Pregnant women with chronic HTN (12–16 weeks gestation)	Early-phase randomized, placebo-controlled feasibility trial	Intervention: L-citrulline (3 g twice daily) Comparator: Placebo	36	High acceptability and good compliance Increased plasma citrulline, arginine, and arginine:ADMA ratio No significant reduction in blood pressure No effect on vascular or angiogenic markers Study underpowered to detect small BP changes
Rodzi et al., 2025 [180]	Malaysia	Patients with hyperglycaemia, HTN, and hyperlipidaemia (3Hs) on standard medications	Randomized, double-blind, placebo-controlled trial	Intervention: Sacha Inchi oil (1000 mg/day) Comparator: Corn oil (placebo)	54	Significant reduction in systolic (−8.6 mmHg) and diastolic BP (−7.0 mmHg) Improved lipid profile (↓ TC, ↓ LDL-C, ↑ HDL-C) No significant effect on glycaemic control Well tolerated with minimal side effects
Toit et al., 2024 [185]	Sweden	Individuals with high blood pressure	Randomized dietary intervention trial	Intervention: Green leafy vegetables (300 mg/day nitrate) and potassium nitrate (PN) supplements Comparator: Potassium chloride placebo with low-nitrate vegetables	70	GLV and PN caused similar nitrate-dependent changes in oral microbiome GLV showed additional beneficial (nitrate-independent) microbiome changes Both increased salivary pH Only GLV improved salivary buffering capacity and reduced lactate GLV may have greater benefit for oral health than PN
Wolak et al., 2019 [179]	Israel	Untreated hypertensive individuals	Randomized, double-blind, placebo-controlled study	Intervention: tomato nutrient complex (TNC) standardized to 5, 15, or 30 mg lycopene Comparators: Synthetic lycopene (15 mg) and placebo	61	Significant reduction in systolic BP observed only with TNC doses of 15 mg and 30 mg lycopene No significant BP effect with 5 mg TNC or 15 mg synthetic lycopene alone Dose-dependent increase in plasma levels of lycopene, phytoene, and phytofluene Higher carotenoid levels (≥15 mg lycopene via TNC) correlated with BP-lowering effect Suggests synergistic effect of carotenoid complex vs. isolated lycopene
Mazza et al., 2018 [177]	Italy	Patients with metabolic syndrome (hypertensive and hypercholesterolemia)	Multicentre, randomized, open-label, post-marketing clinical trial	Intervention: Nutraceutical combination (red yeast rice + Coenzyme Q10) + diet/lifestyle Comparator: Diet/lifestyle alone	104	Greater reduction in systolic BP (−5.2 vs. −3.0 mmHg) and diastolic BP (−4.9 vs. +2.9 mmHg) vs. control Significant improvements in TCl (−17.2%), LDL-C (−21.8%), TG (−16.0%), and glucose (−3.4%) (*p* < 0.001) No significant change in HDL-C No gender-based differences Safe and well tolerated
Nesami et al., 2015 [188]	Iran	Mildly hypertensive patients	Randomized, double-blind, placebo-controlled trial	Intervention: Coenzyme Q10 (100 mg/day) Comparator: Placebo	60	Significant increase in adiponectin levels in CoQ10 group (*p* = 0.04) Significant reduction in IL-6 and hs-CRP levels (*p* ≤ 0.03) No significant change in TNF-α and IL-2 Suggests anti-inflammatory effect of CoQ10 in HTN
Enseleit et al., 2012 [187]	Switzerland	Patients with coronary artery disease	Randomized, double-blind, placebo-controlled crossover trial	Intervention: Pycnogenol (200 mg/day) + standard therapy Comparator: Placebo	23	Significant improvement in endothelial function (↑ FMD: 5.3 → 7.0; *p* < 0.0001) Significant reduction in oxidative stress marker (↓ 15-F2t-isoprostane; *p* = 0.012) No significant change in inflammation markers, platelet adhesion, or blood pressure Suggests endothelial benefits mediated via antioxidant effects
Wada et al., 2020 [190]	Japan	Preschool children (4–5 years) with HTN	Randomized, controlled trial	Intervention: Nori (1.76 g/day) + usual diet Comparator: Usual diet alone	89	Reduction in systolic BP (−8.29 mmHg vs. +0.50 mmHg; borderline significance, *p* = 0.051) Significant reduction in diastolic BP (−6.77 mmHg vs. −0.05 mmHg; *p* = 0.031) Effect observed mainly in boys; no significant change in girls Suggests potential preventive role of seaweed in childhood BP elevation
Mendes et al., 2015 [189]	Brazil	Hypertensive patients (with or without anemia)	Randomized, double-blind, placebo-controlled trial	Intervention: Alpha-lipoic acid (600 mg/day) Comparator: Placebo	60	Significant reduction in total leukocyte count (*p* < 0.05) Increase in neutrophil count Significant reduction in serum iron levels and transferrin saturation index (TSI) Suggests modulation of iron metabolism via metal chelation Potential risk of inducing iron deficiency anemia with supplementation
Abate et al., 2026 [186]	Italy	Adults with grade 1 and grade 2 HTN	Randomized controlled trial	Intervention: Nutraceutical formulation: grape pomace extract (Taurisolo^®^, polyphenol-rich antioxidant) + L-arginine (NO precursor) Comparator: placebo/control		Significant and sustained reductions in systolic and diastolic BP. Improved quality of life (SF-12 questionnaire: vitality, emotional well-being, social functioning). Well tolerated and no major adverse effects.

AS: atherosclerosis; HTN: hypertension; BP: blood pressure; SBP: systolic blood pressure; DBP: diastolic blood pressure; MAP: mean arterial pressure; VO_2_ max: Maximal Oxygen Uptake; NO: nitric oxide; FMD: Flow-Mediated Dilation; TSI: Tissue Saturation Index; HHb: Deoxygenated Hemoglobin; YN: young non-smokers; YS: young smokers; MN: middle-aged subjects; TG: triglycerides; HDL-C: high-density lipoprotein cholesterol; LDL-C: low-density lipoprotein cholesterol; TC: total cholesterol; CETP: Cholesteryl Ester Transfer Protein; PON1: paraoxonase-1; ApoA-I: apolipoprotein A-I; apoB: apolipoprotein B; CBD: cannabidiol; GSE: grape seed extract; PN: potassium nitrate; GLV: green leafy vegetables; TNC: tomato nutrient complex; CoQ10: coenzyme Q10; IL-6: Interleukin-6; IL-2: Interleukin-2; TNF-α: Tumor Necrosis Factor-Alpha; hs-CRP: high-sensitivity C-reactive protein; SF-12: Short Form-12 Health Survey Questionnaire. ↑ indicates increased levels, activation, or upregulation; ↓ indicates decreased levels, inhibition, or downregulation.

Even though the available studies showed promising results, some limitations need to be acknowledged. Several trials were conducted in populations with smaller sample sizes and for shorter durations, limiting the ability to assess long-term benefits. Variability in study design, dosing regimens, and endpoints further complicates direct comparisons across studies. Moreover, many interventions were evaluated as adjuncts to standard therapy, making it difficult to isolate their independent effects.

In conclusion, the available data suggest that nutraceuticals may be useful in the treatment of HTN and CVDs, primarily through metabolic regulation, enhancement of endothelial function, and reduction in oxidative stress. Their broader cardiovascular and metabolic benefits support their incorporation into comprehensive lifestyle-based approaches, even though their blood pressure-lowering effects are often mild. Importantly, variability in clinical response highlights the need to better understand pharmacokinetic factors such as absorption, bioavailability, metabolism, and dose–response relationships. Differences in formulation, dosing, and patient characteristics may significantly influence therapeutic outcomes. Incorporating PK-guided and biomarker-based approaches in future trials could optimize dosing and improve reproducibility. Therefore, long-term, larger randomized controlled trials integrating clinical, biomarker, and pharmacokinetic endpoints are necessary to understand their clinical efficacy, ideal dosage, safety parameters and personalized application of nutraceuticals in the treatment of HTN and CVDs.

## 10. Delivery Strategies of Nutraceuticals and Future Directions

Although several of the nutraceuticals discussed in the previous sections have been shown to demonstrate potent antihypertensive and anti-atherogenic potential, some of their efficacy is still a question due to limited solubility, instability, absorption, and low bioavailability. These limitations can be overcome by the implementation of effective delivery systems to achieve successful maximal bioavailability of desired nutraceuticals. Two widely used delivery systems include emulsions and nanoemulsions. In particular, nanoemulsions (20–200 nm) are used to encapsulate vitamins (A, D, and E), carotenoids (β-carotene, lycopene), curcumin, resveratrol, and CoQ10 to improve water dispersibility, stability, and bioavailability by increasing surface area for digestion [194]. Polymer micelles and nanoemulsions (50–200 nm) can increase the bioavailability of phytochemicals like curcumin and dibenzoylmethane by around three times and achieve up to 85% suppression of inflammation [195]. Nanoemulgels (NEGs) combine nanoemulsions with a gel matrix to improve the solubility, permeability, and skin penetration of actives like ginger extract and 6-gingerol, resulting in higher flux and bioavailability than conventional gels [196]. In contrast to less structured systems, which degrade quickly, emulsion gels exhibit superior long-term stability and can withstand hydrolysis, maintaining over 70% of curcumin [197]. Emulsion gels, on the other hand, can offer better overall exposure, including 44–105% greater EPA/DHA plasma levels than capsules and improved preservation of sensitive active ingredients such as omega-3 fatty acids [197]. Gels and emulsion gels, which are frequently made as chewable or topical medicines, are especially well-suited for nutraceuticals that require protection, such as curcumin and quercetin [197,198].

However, while many systems seem biocompatible at low doses, high concentrations or cationic particles can cause oxidative stress, inflammation, and epithelial damage. Nutraceutical nanoparticles, such as nanoemulsions, solid lipid nanoparticles (SLNs), and liposomes, raise safety concerns due to potential gastrointestinal toxicity, systemic absorption, and long-term accumulation, all of which are strongly influenced by particle size, surface charge, and composition [199]. Important safety issues include interactions with the gastrointestinal tract, where nanoparticles can change the microbiota, tight junctions, and mucus integrity. Food matrices and pH can change the properties of nanoparticles, increasing their reactivity and toxicity; animal models have shown histological results like microvilli atrophy [199]. Because nanoparticles can produce free radicals that harm DNA and lipids and because positively charged particles increase cytotoxicity through strong mucus and epithelial adherence, oxidative stress and inflammation are also crucial [199,200]. Although oral delivery is typically safer than parenteral methods and biodegradable coatings like PEG or chitosan can reduce these concerns, there is currently a lack of reliable long-term human safety data for nutraceutical nanoparticles.

In order to address bioavailability and stability limitations, future trends in nutraceutical formulations will prioritize sophisticated nanotechnology, AI integration, and sustainable plant-based carriers. These include targeted nanocarriers, hybrid systems, and individualized delivery for improved efficacy and safety [201,202]. Innovations in nanotechnology concentrate on hybrid nanoparticles, such as lipid–polymer and chitosan–alginate systems, for the co-delivery of multiple nutraceuticals, enhancing targeted release and facilitating blood–brain barrier crossing for brain health [203]. Other platforms include metal–organic frameworks (MOFs), nanosponges, and dendrimers for controlled release, as well as amorphous FAST-generated nanoparticles (such as curcumin–resveratrol hybrids) with surface charges higher than 50 mV that improve stomach stability [204]. Zein–casein polymer systems and food-grade self-assembling technologies are being developed for scalable, clean-label manufacture [203,204]. New methods include microbiome-modulating nanoprobiotics and AI/ML-optimized formulations to anticipate and improve bioavailability [205]. Sustainable approaches rely on vitamin-specific nanosystems like cyclodextrin–vitamin complexes and plant-based nano delivery systems (NDS) like lignin and pectin carriers [206,207], while human trials of the use of biodegradable materials to reduce toxicity are becoming more and more important in clinical translation efforts [201].

In addition to delivery and safety precautions, several clinically important factors need to be considered. Diverse classes of nutraceutical compounds may interact with conventional cardiovascular medications, for example, statins and anticoagulants, highlighting the importance of close monitoring in clinical studies. Since regulatory frameworks vary across global regions, there is little harmonization in labelling, quality control, and approval procedures, raising concerns about the safety and efficacy of nutraceuticals. The heterogeneity in nutraceutical formulations and absence of standardized dosage regimens makes it difficult to figure out the appropriate dose to take. Therefore, to ensure that nutraceuticals are safe and work well, future research should focus on dosage optimization, global regulatory alignment, and drug–nutraceutical interaction studies.

## 11. Conclusions

Although standard preventive and therapeutic approaches are widely used for the treatment and management of AS and HTN, limitations such as low efficacy and safety concerns persist. Nutraceutical-based approaches are gaining significant interest due to their diverse biological effects. Both preclinical and clinical studies provide valuable mechanistic insights into potential benefits of diverse nutraceuticals (e.g., polyphenols, carotenoids, omega-3 PUFAs, dietary fibers, herbal extracts), including modulation of oxidative stress, inflammation, endothelial dysfunction, vascular smooth muscle cell proliferation, and lipid metabolism.

While nutraceutical bioactive compounds such as curcumin, resveratrol, astaxanthin, and quercetin demonstrate compelling mechanistic effects in preclinical models, clinical evidence remains limited and heterogeneous, with many studies restricted to short-term trials. Additionally, translational applicability is restricted mainly due to poor bioavailability and variability among nutraceutical formulations. Recent advancements in nano-based delivery systems may enhance bioavailability of nutraceuticals and improve clinical outcomes. Dietary fibers and some herbal extracts have been shown to demonstrate moderate clinical evidence in modulating lipid profiles, thereby attenuating AS, and in reducing blood pressure. In contrast, *omega*-3PUFAs are the most clinically proven nutraceutical bioactives with promising antihypertensive and anti-atherosclerotic effects, suggesting their adjunctive role in the management of a range of CVDs.

It is important to note that certain populations (e.g., France and Japan) exhibit significantly lower CVD risk and mortality, indicating the importance of long-term lifestyle practices and balanced diets rich in fish, fruits, vegetables, and antioxidant-rich foods [208,209]. Although the nutraceutical bioactive compounds discussed in this review provide mechanistic insights into AS and HTN, their role should be considered complementary to healthy dietary patterns and lifestyle factors, which remain central to reducing the global cardiovascular burden. Furthermore, socioeconomic factors must also be taken into account, especially in low- and middle-income nations where food scarcity and nutritional deficiencies pose greater challenges than access to nutraceutical supplements.

Overall, nutraceuticals represent a promising adjunct for managing HTN and AS, thereby supporting cardiovascular health. However, their therapeutic potential must be validated through large-scale, well-monitored clinical trials and interpreted with caution.

## Figures and Tables

**Table 1:** Comparative evidence of nutraceuticals in AS.

	Nutraceutical	Mechanistic Evidence	Animal Studies	Clinical Evidence	References
1	Curcumin	Anti-inflammatory, ↓ NF-κB, ↓ ROS	↓ plaque in models	Meta-analysis shows lipid and inflammatory reduction	[141]
2	Resveratrol	Antioxidant, ↑eNOS, ↓ NF-κB	Anti-atherogenic in vivo	Meta-analysis shows ↓ TNF-α	[108]
3	Berberine	↑ LDLR, ↓ PCSK9, ↑ cholesterol efflux	↓ plaque in ApoE^−/−^ mice	Strong lipid-lowering evidence	[142]
4	Beta-glucan	↓ cholesterol absorption, antioxidant	↓ lipid levels	Meta-analysis ↓ LDL-C	[126]
5	Omega-3 (PUFA)	Anti-inflammatory, ↑ RCT	↓ plaque progression	Meta-analysis ↓ CV mortality	[143]
6	Astaxanthin	↑ ABCA1, ↓ MAPK	↓ plaque size	Clinical lipid effects	[144]

**Abbreviations:** NF-κB: nuclear factor kappa-light-chain-enhancer of activated B cells; ROS: reactive oxygen species; eNOS: endothelial nitric oxide synthase; TNF-α: Tumor Necrosis Factor alpha; LDLR: Low-Density Lipoprotein Receptor; PCSK9: proprotein convertase subtilisin/Kexin Type 9; ApoE^−/−^ mice: apolipoprotein E knockout mice; LDL-C: low-density lipoprotein cholesterol; PUFA: polyunsaturated fatty acids; RCT: reverse cholesterol transport; CV: cardiovascular; ABCA1: ATP-Binding Cassette Transporter A1; MAPK: Mitogen-Activated Protein Kinase; ↑ indicates increased levels, activation, or upregulation; ↓ indicates decreased levels, inhibition, or downregulation.

**Table 2:** Comparative evidence of nutraceuticals in HTN.

No.	Nutraceutical	Mechanistic Evidence	Animal Studies	Clinical Evidence	Reference
1	Curcumin	↓ ACE, ↓ Ang II, ↑ NO, ↓ NF-κB, antioxidant	↓ BP in hypertensive rat models	BP reduction and endothelial improvement	[165]
2	Resveratrol	↑ eNOS, ↑ NO, ↓ oxidative stress, anti-inflammatory	↓ BP in hypertensive models	Improved NO-mediated vascular function	[166]
3	Other polyphenols (e.g., caffeic acid and chlorogenic acid)	ACE inhibition, ↑ NO, ↓ ROS, ↓ endothelin-1	↓ BP in experimental models	Clinical/epidemiological evidence shows improved vascular function	[149,167]
4	Bioactive peptides	ACE/renin inhibition, Ang II	↓ BP in SHR	Emerging clinical evidence for antihypertensive effectives	[168]
5	Omega-3 (PUFA)	↓ vascular inflammation, improved endothelial function	↓ BP in animal models	Meta-analysis support for modest BP reduction	[143,169]
6	Polyphenol formulations	↑ NO–cGMP signaling, vasodilation	Experimental suppport	Improved BP and vascular function	[170]
7	Herbal extracts Hibiscus, grape etc)	ACE inhibition, antioxidant, ↑ NO	↓ BP in hypertensive models	Clinical improvement	[171,172]
8	Protein hydrolysates	ACE inhibition, ↑ ACE2 pathway	↓ BP in SHR	Limited clinical	[157,158]

**Abbreviations:** ACE: angiotensin-converting enzyme; Ang II: angiotensin II; NO: nitric oxide; NF-κB: nuclear factor kappa-light-chain-enhancer of activated B cells; BP: blood pressure; eNOS: endothelial nitric oxide synthase; ROS: reactive oxygen species; SHR: spontaneously hypertensive rats; PUFA: polyunsaturated fatty acids; cGMP: cyclic guanosine monophosphate; NOS: nitric oxide synthase; HDL-C: high-density lipoprotein cholesterol; ACE2: angiotensin-converting enzyme 2. ↑ indicates increased levels, activation, or upregulation; ↓ indicates decreased levels, inhibition, or downregulation.

## Data Availability

No new data were created or analyzed in this study. Data sharing is not applicable to this article.

## Abbreviations

The following abbreviations are used in this manuscript:

AS	Atherosclerosis
CVDs	Cardiovascular Diseases
LDL	Low-Density Lipoprotein
LDL-C	Low-Density Lipoprotein Cholesterol
NO	Nitric Oxide
eNOS	Endothelial Nitric Oxide Synthase
LDLR	Low-Density Lipoprotein Receptor
oxLDL	Oxidized Low-Density Lipoprotein
MPO	Myeloperoxidase
ROS	Reactive Oxygen Species
CD36	Cluster of Differentiation 36
SRA-1	Scavenger Receptor Class A-1
ABCA1	ATP-Binding Cassette Transporter A1
ABCG1	ATP-Binding Cassette Transporter G1
NF-κB	Nuclear Factor Kappa-Light-Chain-Enhancer of Activated B s
NLRP3	NOD-, LRR- and Pyrin Domain-Containing Protein 3
IL-1β	Interleukin-1 Beta
VSMC	Vascular Smooth Muscle Cell
PDGF	Platelet-Derived Growth Factor
TGF-β	Transforming Growth Factor-Beta
ECM	Extracellular Matrix
MMP	Matrix Metalloproteinase
TF	Tissue Factor
CO	Cardiac Output
PVR	Peripheral Vascular Resistance
SVR	Systemic Vascular Resistance
SMCs	Smooth Muscle Cells
SNS	Sympathetic Nervous System
RAS	Renin-Angiotensin System
ACE	Angiotensin Converting Enzyme
ApoB	Apolipoprotein B
ASCVD	Atherosclerotic Cardiovascular Disease
PCSK9	Proprotein Convertase Subtilisin/Kexin Type 9
siRNA	Small Interfering RNA
SGLT2	Sodium-Glucose Co-Transporter 2
DASH	Dietary Approaches to Stop Hypertension
PUFAs	Polyunsaturated Fatty Acids
PON1	Paraoxonase-1
RCT	Reverse Cholesterol Transport
NPC1L1	Niemann–Pick C1-Like 1
HNF-1α	Hepatocyte Nuclear Factor-1 Alpha
ApoE	Apolipoprotein E
p38MAPK	p38 Mitogen-Activated Protein Kinase
p16^INK4a	Cyclin-Dependent Kinase Inhibitor 2A
TNF-α	Tumor Necrosis Factor Alpha
ICAM-1	Intercellular Adhesion Molecule-1
VCAM-1	Vascular Cell Adhesion Molecule-1
Nrf2	Nuclear Factor Erythroid 2-Related Factor 2
MKP-1	Mitogen-Activated Protein Kinase Phosphatase-1
ERK1/2	Extracellular Signal-Regulated Kinases 46054
JNK	c-Jun N-Terminal Kinase
COX-2	Cyclooxygenase-2
EZE	Ezetimibe
NC	Nutraceutical Compound
TC	Total Cholesterol
hs-CRP	High-Sensitivity C-Reactive Protein
RYR	Red Yeast Rice
HMG-CoA	3-Hydroxy-3-Methylglutaryl-Coenzyme A
IL-8	Interleukin-8
TLR4	Toll-Like Receptor 4
MYD88	Myeloid Differentiation Primary Response 88
TIRAP	Toll-Interleukin 1 Receptor Domain-Containing Adaptor Protein
TRAF6	TNF Receptor-Associated Factor 6
IRAK1	Interleukin-1 Receptor-Associated Kinase 1
VE-cadherin	Vascular Endothelial Cadherin
ZO-1	Zonula Occludens-1
DGLA	Dihomo-γ-Linolenic Acid
SHR	Spontaneously Hypertensive Rat
MAP	Mean Arterial Pressure
NE	Norepinephrine
IL-6	Interleukin-6
iNOS	Inducible Nitric Oxide Synthase
MCP-1	Monocyte Chemoattractant Protein-1
NOX2	NADPH Oxidase 2
NOX4	NADPH Oxidase 4
AT1	Angiotensin II Receptor Type 1
mAChRs	Muscarinic Acetylcholine Receptors
MDA	Malondialdehyde
L-NAME	Nω-Nitro-L-Arginine Methyl Ester
PCCs	Protein Carbonyl Contents
SOD	Superoxide Dismutase
CAT	Catalase
GR	Glutathione Reductase
GPx	Glutathione Peroxidase
GSH	Reduced Glutathione
Kim-1	Kidney Injury Molecule-1
CTnI	Cardiac Troponin I
RWLP	Rice Wine Lees Peptides
ET-1	Endothelin-1
LRW	Leu-Arg-Trp Tripeptide
ACE2	Angiotensin-Converting Enzyme 2
MasR	Mas Receptor
JAK2	Janus Kinase 2
STAT3	Signal Transducer and Activator of Transcription 3
BNP	B-Type Natriuretic Peptide
MYH7	Myosin Heavy Chain 7
MMP-2	Matrix Metalloproteinase-2
TIMP1	Tissue Inhibitor of Metalloproteinases 1
CTGF	Connective Tissue Growth Factor
APPH	Alcalase Potato Protein Hydrolysate
SP6	Spirulina-Derived Decapeptide (GIVAGDVTPI)
PI3K	Phosphoinositide 3-Kinase
Akt	Protein Kinase B
SHPH	Spent Hen Muscle Protein Hydrolysate
SBP	Systolic Blood Pressure
MABP	Mean Arterial Blood Pressure
LfQR	Limosilactobacillus fermentum Quercetin-Resveratrol Formulation
sGC-cGMP	Soluble Guanylate Cyclase-Cyclic Guanosine Monophosphate Pathway
PK	Pharmacokinetics
